# Delineating spatiotemporal and hierarchical development of human fetal innate lymphoid cells

**DOI:** 10.1038/s41422-021-00529-2

**Published:** 2021-07-08

**Authors:** Chen Liu, Yandong Gong, Han Zhang, Hua Yang, Yang Zeng, Zhilei Bian, Qian Xin, Zhijie Bai, Man Zhang, Jian He, Jing Yan, Jie Zhou, Zongcheng Li, Yanli Ni, Aiqing Wen, Yu Lan, Hongbo Hu, Bing Liu

**Affiliations:** 1 State Key Laboratory of Proteomics, Academy of Military Medical Sciences, Academy of Military Sciences, Beijing, China; 2 State Key Laboratory of Experimental Hematology, Institute of Hematology, Fifth Medical Center of Chinese PLA General Hospital, Beijing, China; 3 Department of Blood Transfusion, Daping Hospital, Army Military Medical University, Chongqing, China; 4 Tianjin Central Hospital of Gynecology Obstetrics, Tianjin, China; 5 Key Laboratory for Regenerative Medicine of Ministry of Education, Institute of Hematology, School of Medicine, Jinan University, Guangzhou, China; 6 Center for Immunology and Hematology, the State Key Laboratory of Biotherapy, West China Hospital, Sichuan University. Collaboration and Innovation Center for Biotherapy, Chengdu, China

**Keywords:** Innate immunity, Haematopoietic stem cells

## Abstract

Whereas the critical roles of innate lymphoid cells (ILCs) in adult are increasingly appreciated, their developmental hierarchy in early human fetus remains largely elusive. In this study, we sorted human hematopoietic stem/progenitor cells, lymphoid progenitors, putative ILC progenitor/precursors and mature ILCs in the fetal hematopoietic, lymphoid and non-lymphoid tissues, from 8 to 12 post-conception weeks, for single-cell RNA-sequencing, followed by computational analysis and functional validation at bulk and single-cell levels. We delineated the early phase of ILC lineage commitment from hematopoietic stem/progenitor cells, which mainly occurred in fetal liver and intestine. We further unveiled interleukin-3 receptor as a surface marker for the lymphoid progenitors in fetal liver with T, B, ILC and myeloid potentials, while IL-3RA^–^ lymphoid progenitors were predominantly B-lineage committed. Notably, we determined the heterogeneity and tissue distribution of each ILC subpopulation, revealing the proliferating characteristics shared by the precursors of each ILC subtype. Additionally, a novel unconventional ILC2 subpopulation (CRTH2^–^ CCR9^+^ ILC2) was identified in fetal thymus. Taken together, our study illuminates the precise cellular and molecular features underlying the stepwise formation of human fetal ILC hierarchy with remarkable spatiotemporal heterogeneity.

## Introduction

Innate lymphoid cells (ILCs), lacking expression of known immune lineage markers and antigen-specific receptors, are a family of lymphocytes as innate equivalents of T cells, and play critical roles in immune response and tissue homeostasis.^1–6^ ILCs have been originally categorized into three, and now into five groups, namely, nature killer (NK) cell, ILC1, ILC2, ILC3 and lymphoid tissue-inducer (LTi) cell.^2,7–12^ Both ILCs and T cells are proposed to be derived from lymphoid progenitors like common lymphoid progenitors (CLPs) and lymphoid-myeloid primed progenitors (LMPPs) in human and mouse, followed by the stepwise commitment to each lymphocyte fate.^13–18^ Commitment to ILC is accompanied by the loss of T cell, B cell and dendritic cell (DC) potentials.^15,19–22^ The final step of ILC maturation is proposed to occur in the tissues where ILCs are located.^23,24^ Transcriptional regulation along ILC development in mouse and human is relatively conservative, involving more than a series of transcription factors (TF). Specifically, ID2, NFIL3, PLZF, TCF1, GATA3, ETS1, TOX, T-BET, EOMES, RORα, BCL11B, AHR and RORγt have been proved to drive commitment, specification and maturation of ILCs from lymphoid progenitors.^19,25–35^

In humans, the ILC progenitors (ILCPs) in circulation are ILC-committed precursors expressing *ID2*, *TOX* and *ZBTB16*. They are identified as Lin^-^CD45^+^CD7^+^CD127^+^CD117^+^CRTH2^-^ phenotype, subsets of which have the capacity to generate all the ILC subsets in vitro and in vivo.^36^ Intriguingly, the ILCPs from human fetal liver predominantly generate ILC3s in co-culture system, suggestive of their remarkable heterogeneity and dynamic spatiotemporal lineage potentials.^36,37^ These precursors might be derived from CD34^+^CD117^+^RORγt^+^ progenitors that also have been found in the second lymphoid tissues. The ILCPs expressing of KLRG1, NKp46 and CD56 were reported to possess different ILC lineage preferences in peripheral blood (PB) and tonsil.^21,38,39^ While some intermediate ILC progenitors/precursors have been described,^36,40,41^ the unbiased hierarchical investigation of ILC differentiation process in the hematopoietic, lymphoid and non-lymphoid tissues in early human development is still unclear. For instance, the intermediate progenitors/precursors along ILC development that bridge hematopoietic stem and progenitors (HSPCs) and committed ILC progenitors remain elusive.

Spatial-temporal distribution and tissue-specific transcriptional heterogeneity of ILCs at single-cell resolution in adult human tissues have been revealed in recent years while those in human fetal tissues remain poorly characterized.^42–45^ Thymus, the primary lymphoid tissue for T cell production, has been recently reported to contain different subsets of ILCs.^46–48^ The LTi-like ILCs, which are important for the development of functional medulla thymic epithelial cells, are readily detectable in human fetal thymus.^49,50^ Importantly, the ILC2-like cells with T cell characteristics are identified in both mouse and human fetal thymus by single-cell RNA-sequencing (scRNA-seq).^46,47^ Consistent with the observation of intrathymic ILC2 development,^51–54^ ILC2 becomes the major ILC subset in the thymus of adult mice.^50^ All these findings pinpoint the potential importance of ILC2 in thymus development and homeostasis.

Here, we provided an unprecedented single-cell transcriptome atlas of stepwise ILC ontogeny in early human fetus using cells from fetal hematopoietic, lymphoid and non-lymphoid tissues spanning 8 to 12 post-conception weeks (PCW). We identified a series of intermediate cell populations along the differentiation paths from human HSPCs to mature ILCs, especially the interleukin-3 receptor alpha positive (IL-3RA^+^) lymphoid progenitors with T, B, ILC and myeloid potentials in fetal liver, combined with computational prediction and functional validation at bulk and single-cell levels. Our study revealed dynamic changes of cell cycle status along with ILC development, from ILC progenitors (quiescent) to the precursors of each ILC subtype (proliferative) and to mature ILCs (quiescent), as well as the heterogeneity and tissue distribution of ILCs. Taken together, our study illuminated the precise molecular events underlying human ILC development at early fetal stage.

## Results

### The spatio-temporal distribution of ILCs in human fetal organs

**Fig. 1:**
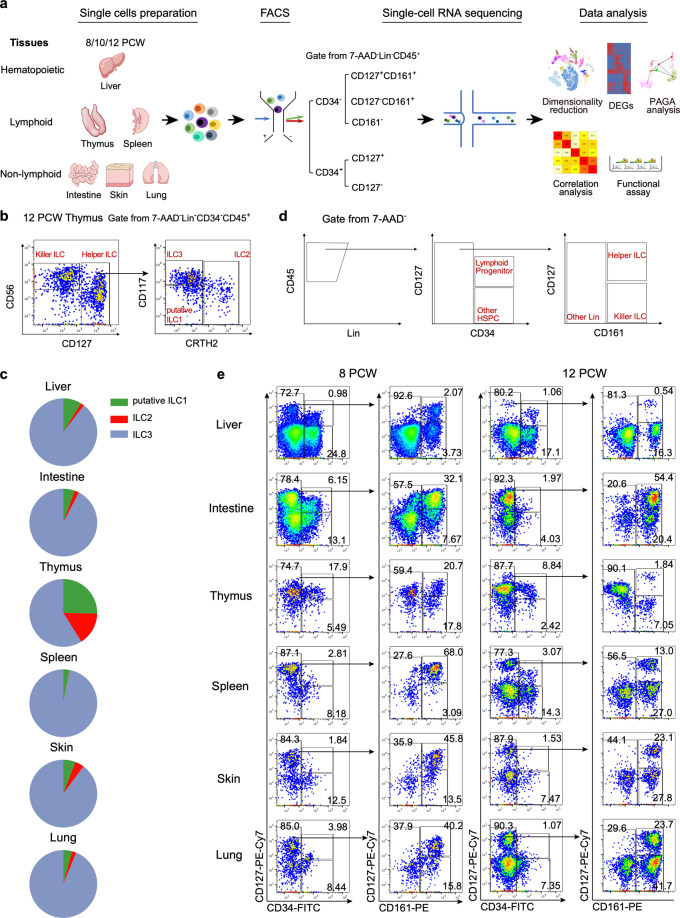
Study strategy and ILC-related populations analysis overview. **a** Schematic overview of the strategy of this study. Cells from human fetal hematopoietic (liver), lymphoid (thymus and spleen) and non-lymphoid (intestine, skin and lung) tissues at indicated gestational stages were first labeled with cell hashing antibodies and fluorescence-labeled antibody for sorting based on the sorting strategy in **e**. The cells from 2–6 tissues were pooled and then loaded on a droplet-based 10× genomics scRNA-seq platform. Cells of each tissue from different samples were distinguished by cell hashing barcode. The information of individual sample was listed in Supplementary information, Fig. S1. Two replicates per developmental stage, with a total of 6 samples were used in this study. **b** The gating strategy to identify human mature ILCs with example from 12 PCW thymus. In the 7AAD^–^ lineage (CD3, CD4, CD5, FcεRI, CD11c, CD11b, CD14 and CD19)-negative (Lin^–^)CD34^-^CD45^+^ cells, there are CD56^+^CD127^–^ killer ILCs and CD56^–^CD127^+^ helper ILC subsets. The helper ILCs are divided into CRTH2^+^ ILC2, CRTH2^–^CD117^+^ ILC3 and CRTH2^–^Kit^–^ putative ILC1. **c** Pie charts show the proportions of three mature helper ILCs of each organ based on flow cytometry analysis. The proportions were calculated based on more than three independent experiments. **d** Sorting strategy of human fetal lymphoid progenitors and ILC-associated populations. In the 7-AAD^–^Lin^–^CD45^+^ cells, CD34^+^CD127^+^ lymphoid progenitors, CD34^+^CD127^–^ HSPCs, CD34^–^CD161^+^CD127^+/–^ ILCs and CD34^–^CD161^–^ cells were sorted from each tissue, and mixed at the ratios indicated in Supplementary information, Fig. S1 for scRNA-seq. **e** The flow cytometry results show that the percentages of the cell populations identified in **d** in each tissue change from 8 to 12 PCW. The results are representative of 2–3 independent experiments in each gestational week.

The development of human ILCs at fetal stage remains to be defined. It has been postulated that they might be from HSPC-derived lymphoid progenitors, which migrate into lymphoid and non-lymphoid tissues for final maturation.^37^ To investigate the molecular basis underlying the human ILC development path from HSPCs to lymphoid progenitors and to each type of ILCs in different tissues at single-cell resolution, we preformed scRNA-seq using cells isolated from the human fetal hematopoietic (liver), lymphoid (thymus and spleen) and non-lymphoid (intestine, skin and lung) tissues at 8, 10 and 12 PCW that are the critical timeframes for ILC development and initiation of lymphoid organogenesis (Fig. 1a; Supplementary information, Fig. S1). The spatiotemporal distribution of mature ILCs and upstream HSPCs and lymphoid progenitors in the fetal tissues were firstly examined by flow-cytometry based on the surface markers indicated in Fig. 1b, c and Fig. 1d, e, respectively. To further confirm the identities of three types of helper ILCs, flow cytometry analysis with co-staining for lineage-specific transcription factors including T-bet, GATA3 and RORγt was conducted (Supplementary information, Fig. S2a). Among Lin^–^CD45^+^CD127^+^ ILCs, CRTH2^+^ and CD117^+^ ILCs showed the highest expression level of GATA3 and RORγt, indicating their conventional ILC2 and ILC3 identities, respectively. As expected, the highest expression of T-bet was detected in Lin^–^CD45^+^CD127^–^CD56^+^ cells, suggestive of killer ILCs. The CRTH2^–^CD117^–^ ILCs were annotated as “putative ILC1 cells”, based on the lack of conventional CD127^+^ ILC1-specific markers.^1^ These cells showed heterogeneous expression of T-bet, GATA3 and RORγt, suggesting that they were heterogeneous, containing classical ILC1s and non-classical ILC2s and ILC3s. Within lineage (CD3, CD4, CD5, FcεRI, CD11c, CD11b, CD14 and CD19)-negative (Lin^–^)CD34^–^CD45^+^ cells, CD56^+^CD127^–^ cytotoxic ILCs, and CD56^–^CD127^+^ helper ILCs that were further divided into CRTH2^+^ (ILC2), CRTH2^–^CD117^+^ (ILC3) and CRTH2^–^CD117^–^ (putative ILC1) were found in all tested tissues (Fig. 1c; Supplementary information, Fig. S2b). The percentages of putative ILC1 and ILC2 in thymus were much higher than those in other tissues. Intriguingly, ILC3 was the dominate population in all tissues except for thymus, accounting for over 90% of Lin^–^CD45^+^CD127^+^ ILCs (Fig. 1c). The absolute cell numbers of putative ILC1 and ILC2 in thymus were also higher than those in other tissues except for intestine (Supplementary information, Table S1). In addition, most, if not all, CRTH2^+^ ILC2 in skin were CD117 positive, which was different from those in other tissues (Supplementary information, Fig. S2b). NKp44, the important surface marker of ILC3,^10^ became detectable around week 12, mostly restricted to ILC3 in intestinal and lung, which was consistent with previous studies that the majority of ILC in the fetal small intestines express both CD69 and NKp44 from week 12 onward and ILC3s in fetal livers at 6 to 20 PCW were mainly NKp44^−^^55–57^ (Supplementary information, Fig. S2c).

As expected, fetal liver, the predominant organ of fetal hematopoiesis at the range of our study,^58^ had the largest proportion of HSPCs (Lin^–^CD45^+^CD34^+^CD127^–^) (Fig. 1d, e; Supplementary information, Fig. S3a, b). The percentage of lymphoid progenitors (Lin^-^CD45^+^CD34^+^CD127^+^) in fetal thymus was the highest among all the examined tissues, which presumably contained T/ILC progenitors.^46,51,53,59–62^ Meanwhile, our data showed that fetal intestine had a considerable proportion of lymphoid progenitor at week 8, which was consistent with the previous studies indicating that intestine is an ILC-rich organ at fetal and adult stages^23,63^ (Fig. 1e; Supplementary information, Fig. S3a, b). The percentage of lymphoid progenitors in intestine reduced dramatically at 12 PCW, correlated with the increased CD161^+^CD127^+/–^ ILCs, presumably due to the formation of mesenteric lymph nodes (mLNs) in the beginning of the second trimester of pregnancy.^8^ Interestingly, the frequency of CD161^+^CD127^+^ ILCs, mostly representing the helper ILCs, dramatically reduced in thymus from 8 to 12 PCW (20.7% to 1.98%, of Lin^-^CD45^+^CD34^–^ cells) (Fig. 1e; Supplementary information, Fig. S3a, b), which was in accordance with the dynamics of ILC in the previous studies.^46^ Thus, our study reveals the spatio-temporal distribution of lymphoid progenitor and ILCs in human fetal organs.

After confirming the presence of mature ILC and potent upstream progenitor or precursor cells in hematopoietic, lymphoid and non-lymphoid tissues, we labeled the cells from each tissue of individual embryo with hashtag oligos (HTO) before fluorescence-activated cell sorting (FACS) analysis. Within Lin^–^CD45^+^ cells, HSPCs (CD34^+^CD127^–^), lymphoid progenitors (CD34^+^CD127^+^), CD161^+^ ILCs (CD34^–^CD127^+/–^CD161^+^) as well as CD3^–^CD161^–^ cells were sorted and mixed in the ratios indicated in Supplementary information, Fig. S1. To avoid genetic bias, two embryos were used for each time point. Cells from tissues were then pooled for 10× genomics scRNA-seq (Fig. 1a). After quality control and doublets exclusion, a total of 31,233 cells were captured, including 9477 cells from liver, 5105 cells from intestine, 4220 cells from thymus, 2112 cells from spleen, 4868 cells from skin and 5451 cells from lung (Supplementary information, Fig. S4a–c). The average number of genes and transcripts per cell was 2,837 and 12,982, respectively (Supplementary information, Fig. S4d, e).

### Single-cell transcriptomic profiling of lymphoid-related populations in human fetus

**Fig. 2:**
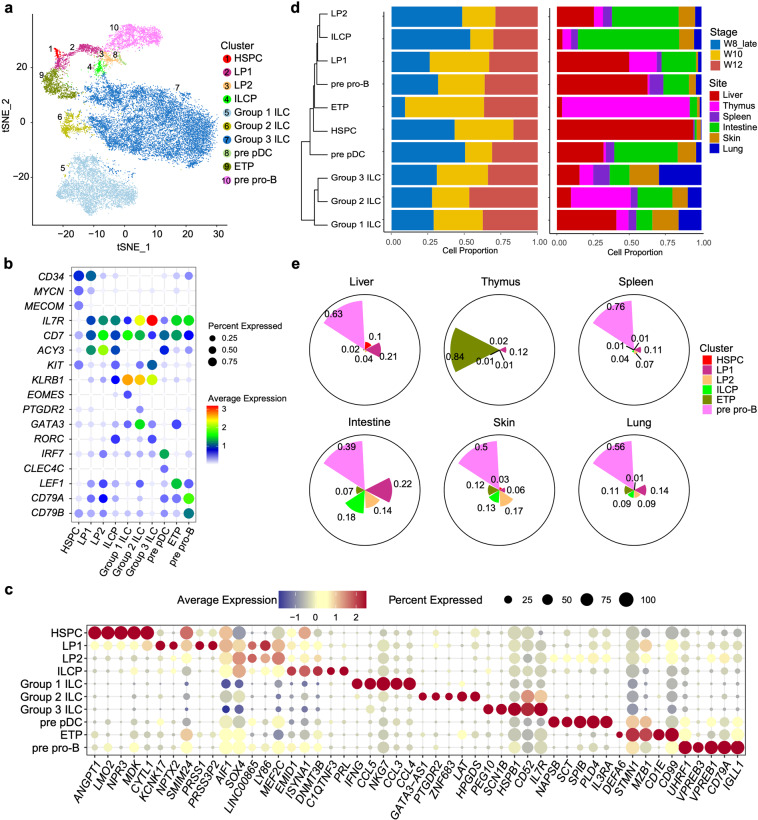
Single-cell transcriptome profiling of lymphoid-related population in human fetal hematopoietic, lymphoid and non-lymphoid tissues. **a***t*-SNE visualization of ten lymphoid-related cell clusters on the scRNA-seq data of cells isolated from human fetal hematopoietic (liver), lymphoid (thymus and spleen) and non-lymphoid (intestine, skin and lung) tissues at 8,10 and 12 PCW. Color indicates the cell identity. **b** Dot plots show the featured genes expressed in different cell clusters. Colors represent the average gene expression levels, and size encodes the proportion of gene-expressing cells. **c** Dot plots show the expression level of the top 5 DEGs of each cell cluster. Colors represent the average expression and size encodes the proportion of gene-expressing cells. **d** Hierarchical clustering of lymphoid-related cell clusters based on Euclidean distance (left panel). The dynamic change of these populations during embryonic development (middle panel) and their tissue distribution (right panel) are shown in bar graph. Embryonic stages and tissues are indicated by colors. **e** Pie charts show the proportions of six progenitor clusters in each organ. The radii of sectors indicate proportions of each cell cluster.

To facilitate the delineation of lymphoid-related populations, we removed the unrelated hematopoietic lineages such as granulocyte-macrophage progenitors, megakaryocyte-erythroid progenitors, and their progeny cells, together with the contaminated non-hematopoietic cells, including epithelial, endothelial and mesenchymal cells (Supplementary information, Fig. S5a, d and Table S2). For the same purpose, only the most primitive B cell progenitors (pre pro-B cells), and T cell progenitors (early thymic progenitor, ETP) were included for further analyses (Supplementary information, Fig. S5a–e). We identified ten lymphoid-related clusters according to the expression of curated genes (Fig. 2a, b; Supplementary information, Table S3). HSPC cluster was distinguished by the enrichment of stem and progenitor cells-related genes, such as *CD34*, *MYCN* and *MECOM*. Two lymphoid progenitor (LP) clusters, LP1 and LP2, were readily identified based on the expression of lymphoid-specific genes *CD7* and *IL7R*. The expression level of *CD34* in LP1 was higher than in LP2, suggesting that LP1 might be upstream of LP2. In addition to *CD7* and *IL7R*, B-lineage genes *CD79A* and *CD79B* were highly expressed in pre pro-B cells. Meanwhile, enrichment of *GATA3* and *TCF7* in ETP cluster was in line with its T-lineage potential. Precursors of plasmacytoid dendritic cells (pre pDC) were characterized by the expression of *IRF7, CLEC4C* and remaining expression of *CD34*.

*IL7R* and *CD7* were also highly expressed in ILCPs and three ILC populations (Group 1, 2, 3 ILC), consistent with the flow cytometry results. ILCPs expressed low level of *CD34*, indicative of their progenitor identity, and the expression of *KLRB1* (encoding CD161) was also lower than mature ILCs (Fig. 2b). Importantly, *RORC*, encoding the essential TF (RORγt) for all ILCs and especially ILC3 development,^21,30^ was enriched in ILCP cluster. Consistent with the expression of cell surface marker, Group 1 ILC showed lower expression of *IL7R* and *KIT*, but higher expression of *KLRB1*, than other ILC groups. The identification of Group 1 ILC was also confirmed by the enriched *EOMES*, which encodes the signature TF for cytotoxic ILC, and *IFNG* (Fig. 2b, c). Group 2 ILC cluster was annotated based on the expression of *GATA3* and *PTGDR2* (encoding CRTH2).^64^ Group 3 ILC was featured by the expression of *RORC*, and the highest expression level of *IL7R* and *KIT*. Hierarchical clustering analysis showed that LP2 and ILCP were closely located (Fig. 2d), indicating a strong correlation between these populations. Group 1 ILC, consisting of NK cells, were separated from Group 2 ILC and Group 3 ILC, suggesting the developmental branch of cytotoxic and helper ILCs as discovered in mouse^65–67^ (Fig. 2d).

The spatial distribution of these cells revealed by the scRNA-seq data was largely in line with the flow cytometry results. The majority of HSPCs were located in fetal liver, meanwhile fetal liver also contained two types of LPs and pre pro-B cells, reconciling with its function as the niche for early development of lymphoid progenitors and B cells. Notably, the proportion of LP1 was higher than that of LP2 in fetal liver, but more LP2s and ILCPs were found in intestine, collectively suggesting that intestine as the ILCs-rich organ which might provide the optimized microenvironment for ILCs development (Fig. 2d, e; Supplementary information, Fig. S5f). The majority of ETPs resided in thymus. 41% of ILCs in thymus were ILC2, which was consistent with the flow cytometry data (Fig. 1b, c; Supplementary information, Fig. S2b). Taken together, our scRNA-seq data revealed the cellularity and tissue distribution of HSPCs, lymphoid progenitors and ILCs at the end of the first trimester of pregnancy.

### The IL-3RA as the marker of lymphoid progenitors with T, B, ILC and myeloid potential in human fetal liver

**Fig. 3:**
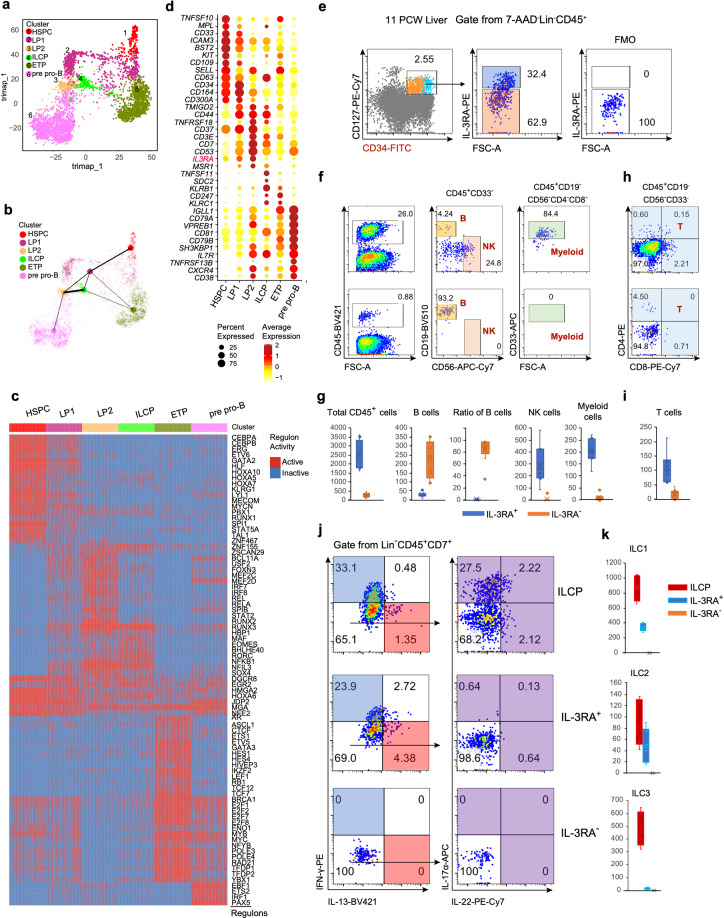
Validation and lineage potential assessment of human CD34^+^CD127^+^IL-3RA^+^ and CD34^+^CD127^+^IL-3RA^–^ lymphoid progenitors in fetal liver. **a** Visualization of HSPC, LP1, LP2, ILCP, ETP and pre pro-B cell clusters by trimap assay. **b** Partition-based graph abstraction (PAGA) topology tree of cell clusters. The width of line in PAGA indicates the strength of connectivity between cell clusters. The colors represent cell identities in **a** and **b**. **c** Heatmap shows the highly expressed TF-encoding genes in progenitor clusters. **d** Dot plots show the expression level of top 10 DEGs in HSPC, LP1, LP2, ILCP, ETP and pre pro-B cell cluster in liver. Colors represent the gene expression level and size encodes the proportion of gene-expressing cells. *IL3RA* highlighted in red is highly expressed in LP1 and LP2 cells. **e** Flow cytometry plots show stronger CD34 expression in IL-3RA^+^ than that in IL-3RA^–^Lin^–^CD45^+^CD34^+^CD127^+^ lymphoid progenitors of human fetal liver. Gating strategy for IL-3RA was based on Fluorescence Minus One (FMO) control (right panel). **f**, **h** IL-3RA^+^ and IL-3RA^–^Lin^–^CD45^+^CD34^+^CD127^+^ lymphoid progenitors (150 cells for each subset) were sorted and cocultured with SCF, FL, IL-7 and IL-15 on OP9 stromal cells (**f**) and OP9-DL4 stromal cells (**h**). The IL-3RA^+^ lymphoid progenitors generate more CD45^+^ hematopoietic cells and have more NK, myeloid (upper panel in **f**) and T lineage (upper panel in **h**) potential while IL-3RA^–^ lymphoid progenitors show B lineage commitment (below panel in **f**) suggested by coculture experiments. B, NK, myeloid and T cells were identified as positive for its respective lineage markers and negative for other lineage markers. **g**, **i** Box plots show the numbers of indicated lineage cells, and the percentage of B cells in total CD45^+^ hematopoietic cells after cocultured for 8 days on OP9 stromal cells (**g**) or 12 days on OP9-DL4 stromal cells (**i**). **j** Fetal liver ILCP (Lin^–^CD45^+^CD7^+^CD127^+^CD117^+^, upper panel), IL-3RA^+^ (middle panel) and IL-3RA^–^Lin^–^CD45^+^CD34^+^CD127^+^ lymphoid progenitors (below panel) were cultured on OP9-DL4 stromal cells for 10–14 days under ILC-induction condition (IL-2, IL-7, IL-1β, IL-23, IL-25 and IL-33). After stimulation with PMA/ionomycin 4 h, cytokine production was analyzed by flow cytometry analysis. IL-17α and IL-22 producing cells were analyzed after gating on cells negative for IFN-γ or IL-13. **k** Box plots show numbers of ILC1, ILC2 and ILC3 production of the indicated populations. ILC1, ILC2 and ILC3 were identified as shown in **j**.

To delineate the lymphocyte lineage differentiation in human fetal liver at early stage, the progenitor populations identified above were selected for further analysis. Partition-based graph abstraction (PAGA) analysis was used to demonstrate their topological location of developmental trajectories, revealing their putative developmental orientation (Fig. 3a, b). The result showed HSPC was at the start point, directly followed by LP1, accompanied by the downregulation of stem and progenitor-related TF-coding genes, like *CEBPA*, *ERG*, *HLF*, *MECOM* and *SPI1*^68^ (Fig. 3a–c). Consistently, few HSPCs displayed a lineage-primed state predicted by STEMNET analysis (Supplementary information, Fig. S6a, b and Table S4). On the other hand, the lymphoid lineage (including T, B and ILC cells) potentials of LP1 was predicted, although the majority of LP1s were still un-committed since these cells were located in the central position of the STEMNET map. *CEBPA* and *MECOM* were further downregulated in LP2s (Fig. 3c). LP2s were likely downstream of LP1 based on the PAGA results, and they might have divergent development paths, including myeloblast and pDC (mature pDC: GSE143002^69^) paths as well as T, B and ILC paths (Fig. 3b; Supplementary information, Fig. S6a, b). More importantly, *NFIL3*, transitionally expressed before *ID2* that is important for ILC commitment, was initially upregulated in LP2 and sustained its expression in ILCP.^27^ (Fig. 3c). ILCP, showing the strongest connection with LP2 (Fig. 3b), was featured by the enrichment of ILC specification-related genes, such as *RORC* and *EOMES*. High expression of *RUNX3* in ILCPs suggested their differentiation potentials to both cytotoxic and helper ILCs especially ILC3.^70,71^ As anticipated, the pre pro-B were restricted to B-cell lineage, with upregulation of B-cell feature TFs-encoding genes including *EBF1* and *PAX5*. ETP, highly expressing *GATA3*, *HES1* and *MYB*, showed both T and ILC potentials, which was consistent with the previous findings showing that the uncommitted Lin^–^CD34^+^CD1a^–^ human thymic progenitors could develop into ILCs^51^ (Fig. 3c; Supplementary information, Fig. S6a, b).

Our previous study has shown that *IL3RA* is overrepresented in the presumed thymus seeding progenitors (TSPs) in human fetal liver.^46^ In the present study, we found that *IL3RA* was also enriched in LPs (LP1 and LP2), suggesting the potential function of IL-3RA on ILC lineage decision (Fig. 3d). By flow cytometric analysis, we found that about one third of immunophenotypically identified lymphoid progenitors (Lin^–^CD45^+^CD34^+^CD127^+^) in fetal liver were IL-3RA^+^. The stronger CD34 expression on IL-3RA^+^ lymphoid progenitors indicated the higher hierarchy and more lineage potential of IL-3RA^+^ lymphoid progenitors than those of IL-3RA^–^ cells (Fig. 3e). We then explored the lineage differentiation potential of the IL-3RA^+^ and IL-3RA^-^ lymphoid progenitor cells in fetal liver by co-culture with the OP9 and OP9-DL4 stromal cells (Fig. 3f–k; Supplementary information, Fig. S6c–f). For differentiation assay at bulk level, IL-3RA^+^ progenitors generated ten-fold more CD45^+^ hematopoietic cells than IL-3RA^–^ progenitors coculturing with OP9 or OP9-DL4 stromal cell with IL-7, SCF, FL and IL-15, suggesting the stronger differentiation capacity of IL-3RA^+^ cells (Fig. 3f, g; Supplementary information, Fig. S6c, d). Intriguingly, IL-3RA^–^ progenitors generated more CD19^+^ B cells than IL-3RA^+^ progenitors (Fig. 3f, g; Supplementary information, Fig. S6c, d). Moreover, IL-3RA^–^ progenitors seemed to be largely B cell lineage-committed since majority of the cocultures with IL-3RA^–^ progenitors (6 wells of total 9 wells in two independent experiments) generated only B cells (Fig. 3f, g). However, all the cocultures with IL-3RA^+^ progenitors could generate multiple lineage cells, including CD33^+^ myeloid cells and CD56^+^ NK cells besides B cells (Fig. 3f, g). In the OP9-DL4 coculture system, the B lineage differentiation capacity of both lymphoid progenitors was dramatically inhibited by NOTCH signaling, but IL-3RA^+^ lymphoid progenitors generate more T cells (Fig. 3h, i) as well as NK cells and myeloid cells (Fig. 3h, i; Supplementary information, Fig. S6c, d). Collectively, our functional data indicated that IL-3RA^+^ progenitors had multiple lineage potentials and IL-3RA^–^ progenitors were mainly B cell committed.

To further confirm the ILC potential of IL-3RA^+^ and IL-3RA^–^ progenitors, we used lymphoid progenitor cells (Lin^–^CD45^+^CD7^+^CD127^+^CD117^+^) isolated from fetal liver as positive control for ILC-induction coculture experiment. These cells contained ILCP identified in our study (Fig. 3j), and were also phenotypically consistent with the findings from Di Santo’s group.^36^ Under the ILC-induction condition (in the presence of IL-2, IL-7, IL-1β, IL-23, IL-25 and IL-33), ILCP generated IFN-γ-producing ILC1, IL-13-producing ILC2 and IL-17/IL-22-producing ILC3 within Lin^–^CD45^+^CD7^+^ cells consistent with the definition used by Di Santo’s group (Fig. 3j). Meanwhile, IFN-γ-producing ILC1 cells and IL-13-producing ILC2s were generated by IL-3RA^+^ lymphoid progenitors, and a few IL-17/IL-22-producing ILC3s were also detected in the IL-3RA^+^ cell differentiation products (Fig. 3j, k). More importantly, compared with IL-3RA^+^ cells, IL-3RA^–^ cells showed much less hematopoietic cell production and IFN-γ, IL-13, IL-17α and IL-22-producing cells were hardly observed, which further indicates the lack of helper ILC lineage potential of IL-3RA^–^ lymphoid progenitors (Fig. 3j, k).

Single-cell clonal assay showed that IL-3RA^+^ lymphoid progenitors represented a heterogeneous population of uni-potent and multipotent progenitors. 7% of the cultures derived from single IL-3RA^+^ progenitors generated only NK cells whereas the remaining cells were multipotent progenitors that could give rise to two or more lineages. Approximately 20% of the cultures could generate CD19^+^ B cells, CD4^+^ and/or CD8^+^ T cells, CD56^+^ NK cells and CD33^+^ myeloid cells (Supplementary information, Fig. S6e). Under ILC-induction condition, IFN-γ-producing ILC1 cells, IL-13-producing ILC2s and IL-17α and/or IL-22-producing ILC3s were generated by most of single-cell cocultures with IL-3RA^+^ lymphoid progenitor cells. The cell numbers of ILC2 and ILC3 were low, possibly due to the limited ILC induction efficiency of lymphoid progenitors that were not committed to ILC yet. Therefore, the results of ILC-induction at single-cell level were consistent with the functional data at bulk level (Fig. 3j, k; Supplementary information, Fig. S6f).

In summary, our functional data at bulk and single-cell levels indicate the difference of lineage potential between IL-3RA^+^ and IL-3RA^–^CD45^+^CD127^+^CD34^+^ lymphoid progenitors. IL-3RA^+^ lymphoid progenitors showed multi-potent differentiation potentials to T, B, ILC and myeloid lineage, while IL-3RA^–^ cells were mainly committed to B lineage.

### Heterogeneity and tissue distribution bias of Group 1 ILCs

**Fig. 4:**
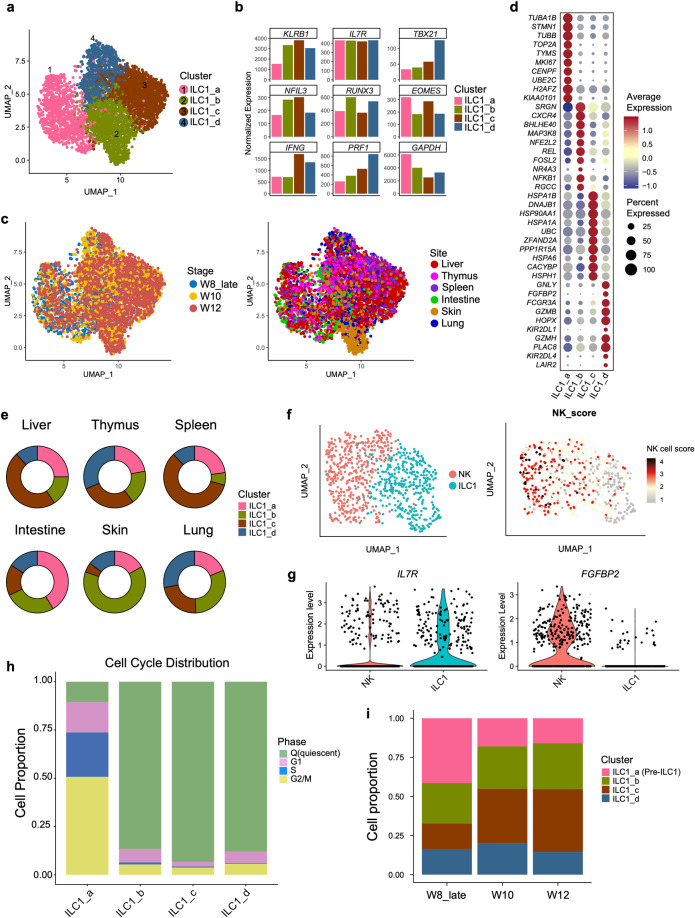
Heterogeneity and tissue distribution bias of Group 1 ILCs. **a** UMAP visualization of Group 1 ILCs from Fig. 2a with re-clustered populations mapped on. Colors indicate cell types. **b** Expression of the known ILC1-related genes in the different ILC1 sub-clusters is shown in bar graph. **c** Stage (left panel) and site (right panel) information of ILC1 sub-clusters identified in **a**, indicated by colors. **d** Dot plots show top 10 DEGs in the ILC1_a, ILC1_b, ILC1_c, ILC1_d clusters. Colors represent gene expression levels, and size encodes the proportion of gene-expressing cells. **e** Pie charts show the proportions of ILC1_a, ILC1_b, ILC1_c and ILC1_d in tissues. Colors represent cell clusters described in **a**. **f** UMAP visualization of the cells in ILC1_d cluster are further separated into NK and ILC1 subgroup by UMAP analysis (left panel). NK score (GEO: GSE70580**)** is projected onto UMAP visualization, and colors indicate the expression level of NK-related genes (right panel). **g** Violin plots show the expression level of *IL7R* and *FGFBP2* in NK and ILC1 subgroups defined in **f**. **h** The proportions of cell in different cell cycle statues in ILC1 sub-clusters are shown in bar graph. Colors indicate different cell cycle statuses. **i** The proportions of ILC1 sub-clusters in each embryonic development stages are shown in bar graph. Colors indicate cell types.

To delineate the heterogeneity of Group 1 ILC, we further divided these cells into four sub-clusters, from ILC1_a to ILC1_d (Fig. 4a). Group 1 ILC-featured genes, *TBX21*, *NFIL3*, *EOMES* and *IFNG* were highly expressed in all ILC1 sub-populations, suggestive of their identities (Fig. 4b; Supplementary information, Table S5). Interestingly, our data showed the tissue-preferred distribution of ILC1 sub-clusters, especially of ILC1_b. Cells in the ILC1_b cluster were largely from skin, particularly from the skin of 10 and 12 PCW, probably indicative of the specialized ILC1 state in skin at this stage (Fig. 4c). *CXCR4*, the gene encoding the receptor of chemokine CXCL12 (SDF-1) expressed on the primary human skin fibroblast,^72,73^ was enriched in ILC1_b cluster that might be involved in the recruitment of ILC1_b to skin (Fig. 4d). Whether CXCL12-CXCR4 interaction also plays a role in ILC1_b development remains elusive. ILC1_c cells were preferably located in liver and spleen (Fig. 4e), with high expression of molecular chaperone-coding genes such as *HSPA1B*, *HSP90AA1* and *HSPA1A*, and their function needs further investigation.

Although no distinct NK population was identified in our study due to the universal expression of *EOMES* in Group 1 ILC cells (Fig. 4b), cytotoxicity-related genes, *FGFBP2*, *FCGR3A* (encoding CD16a) and *GZMB*, were enriched in ILC1_d cluster, highlighting their NK and/or ILC1 identities (Fig. 4d). Further analysis revealed the heterogeneity of ILC1d cells that contained NK and ILC1 subsets, confirmed by curated NK genes scoring (GEO: GSE70580, Fig. 4f). In accordance with our previous study, *IL7R* was enriched in ILC1 subset, but not in NK cells, and *FGFBP2* showed restricted expression in the NK subset (Fig. 4g).

ILCs are considered as the tissue-resident lymphoid cells in mouse and human. At adult stage, the ILC precursors in the secondary lymphoid organs are proposed to give rise to local ILCs, while the developmental event at fetal stage is largely unknown. The cell cycle analysis showed that most of ILC1s were quiescent except for those in ILC1_a (Fig. 4h; Supplementary information, Table S6). The highly proliferative feature of ILC1_a was also evidenced by the high expression of *MKI67* and *TOP2A*,^74^ the latter gene encoding DNA topoisomerase that regulates the DNA topologic states during DNA replication (Fig. 4d). Given the low expression level of *KLRB1*, we hypothesized that these cells might be the ILC1 precursors (Fig. 4b). Among all the Group 1 ILC subsets, the proportion of ILC1_a was the highest at week 8, and then reduced at week 10 and 12, indicating that the development of ILC1_a might occur earlier than other ILC1 subsets (Fig. 4i). Like LP2 and ILCP, ILC1_a cells were enriched in intestine, highlighting the critical role of intestine in ILC development (Fig. 4e). As expected, the predicted putative trajectory started from ILC1_a cells (Supplementary information, Fig. S7a, b), further indicating its precursor identity, followed by three main different development routes toward ILC1_d, ILC1_b and ILC1_b to ILC1_c, respectively. Taken together, our study unveils the heterogeneity of ILC1 and potential ILC1 precursors.

### The heterogeneity of conventional and unconventional ILC2 subsets

**Fig. 5:**
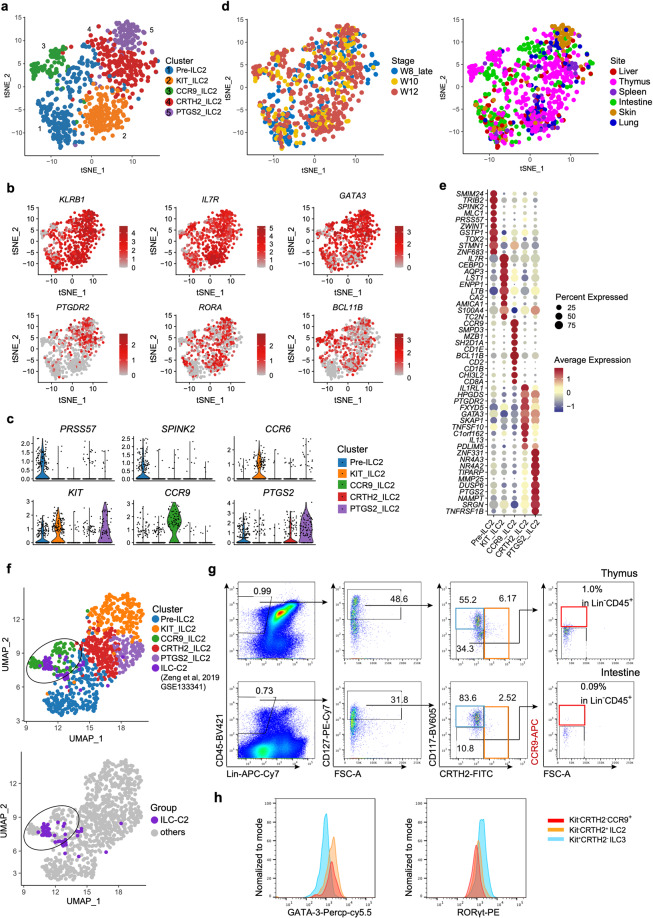
The heterogeneity of conventional and unconventional ILC2 subsets. **a** UMAP visualization of Group 2 ILC with five subpopulations. Colors indicate cell types. **b** Expression of the indicated genes projected to UMAP of ILC2 sub-clusters. Colors indicate the scaled gene expression level. **c** Violin plots show the expression of featured genes in ILC2 sub-clusters. Colors represent cell clusters. **d** UMAP visualization of ILC2 sub-clusters described in **a**. Stage (left panel) and site (right panel) information of different ILC2 sub-clusters indicated by colors. **e** Dot plots show the top 10 DEGs in Pre_ILC2, Kit_ILC2, CCR9_ILC2, CRTH2_ILC2, PTGS2_ILC2 clusters. Colors represent the average expression and size encodes the proportion of gene-expressing cells. **f** ILC-C2 cluster in human fetal thymus identified previously (GEO: GSE133341) is projected to UMAP of ILC2 sub-clusters. ILC-C2 cells are highlighted and the ILC2 cells in this study are in gray (below panel). **g** Representative flow cytometry results show the frequencies of CCR9_ILC2 in fetal thymus (upper) and intestine (below). CCR9_ILC2 cell is gated as Lin^–^CD45^+^CD127^+^CD117^–^CRTH2^–^CCR9^+^. **h** Flow cytometry results show the expression of GATA-3 and RORγt in CD117^–^CRTH2^–^CCR9^+^ ILC2 (red), conventional CRTH2^+^ ILC2 (orange) and CD117^+^CRTH2^–^ ILC3 (blue) cells.

The heterogeneity of Group 2 ILC was also investigated. The cluster was separated into five sub-clusters (Fig. 5a; Supplementary information, Table S5), all of which were characterized by the high expression of ILC2-featured TF coding genes, *GATA3* and *RORA* (Fig. 5b). Cells in Pre-ILC2 sub-cluster were proposed to be ILC2 precursors, based on the enrichment of *PRSS57* and *SPINK2*, as well as the reduction of proportion from 8 to 12 PCW (Fig. 5c; Supplementary information, Fig. S8a). Pre-ILC2 were further sub-grouped into Pre-ILC2a and Pre-ILC2b, both of which expressed *SPINK2* and *PRSS57* while proliferating-related gene *MKI67* and ILC2-specialized genes *PTGDR2* and *BCL11B* were enriched in the latter one (Supplementary information, Fig. S8b, c), indicating that Pre_ILC2b might be derived from Pre_ILC2a. It was conceivable that Pre_ILC2a has multipotent capability, which needs to be further deciphered by functional experiments. Two conventional ILC2 sub-clusters, CRTH2_ILC2 and PTGS2_ILC2, were identified. Both of them expressed high level of *PTGDR2* (encoding CRTH2), while the latter specifically overrepresented *PTGS2* that is essential for human ILC2 activation in PB and tonsil (Fig. 5b, c).^75^ Highest expression of *IL1RL1* (encoding IL-33 receptor) and *IL13* in CRTH2_ILC2 (Fig. 5c, e) indicated their mature status. Enrichment of *NR4A2* and *NR4A3* suggested that PTGS2_ILC2 might be transcriptionally correlated with the newly identified ILC2 subpopulation localized with the fibroblast-like adventitial stromal cells^76^ (Fig. 5e). Its skin-resident characteristics together with expression of *KIT* are consistent with the previous flow cytometric analysis (Fig. 5c, d; Supplementary information, Fig. S8d). Two unconventional ILC2 subsets with low *PTGDR2* expression (KIT_ILC2 and CCR9_ILC2), were also identified (Fig. 5a–c). Considering the recently reported c-Kit^hi^CCR6^+^ ILC2 subset which can convert into IL-17-producing NKp44^−^ ILC3-like cells,^77,78^ we hypothesized that KIT_ILC2 cells which also expressed high level of *CCR6* and *LTB*, might be the fetal counterparts of the c-Kit^hi^CCR6^+^ ILC2 cells with ILC2-ILC3 plasticity.

CCR9_ILC2 was featured by the enrichment of *BCL11B* and *CCR9* (Fig. 5b, c). BCL11B is the critical TF for both T cell commitment^79^ and maintenance of the genetic and functional programs of ILC2,^26,80,81^ and CCR9 is essential for intrathymic seeding of T progenitors.^82–84^ Intriguingly, majority of these cells were from thymus, which might be due to the CCR9–CCL25 interaction. In a previous study, we have identified a unique ILC population (ILC-C2) in human fetal thymus (GEO: GSE133341),^46^ showing the high expression of T cell-featured genes (*CD3G* and *CD4*), and ILC2-related genes (*GATA3* and *IL4)*. Expression of *CD1E, CD2* and *CD8A* in CCR9_ILC2 also suggested its close relationship with T cells (Fig. 5e). After being projected to UMAP with all Group 2 ILC cells from the present study, these ILC-C2 cells were clustered with CCR9_ILC2, further validating their ILC2 identity (Fig. 5f). To further examine whether this unique CCR9_ILC2 subpopulation could be captured at protein level, we performed flow cytometric analysis and found that among the Lin^–^CD45^+^CD127^+^CD117^–^CRTH2^–^ cells in human fetal thymus and intestine, there was a minor CCR9^+^ population in both tissues that was phenotypically consistent with CCR9_ILC2 identified by the transcriptome data, with relatively higher proportion in thymus (Fig. 5g). Next, we exanimated the expression of ILC-specific TFs of this population by flow cytometry. Similar to the conventional ILC2s (Lin^–^CD45^+^CD127^+^CD117^–^CRTH2^+^), CCR9^+^ ILC2 had higher expression of GATA3 but lower expression of RORγt than ILC3 cells (Lin^–^CD45^+^CD127^+^CD117^+^CRTH2^–^) (Fig. 5h). Taken together, using transcriptomic and immunophenotypic analysis, we find a new CCR9^+^PTGDR2^–^ ILC2 population at fetal stage.

### Computational prediction of LTi-like cell and Non-LTi ILC3 pathways from proliferating ILC3 precursors

**Fig. 6:**
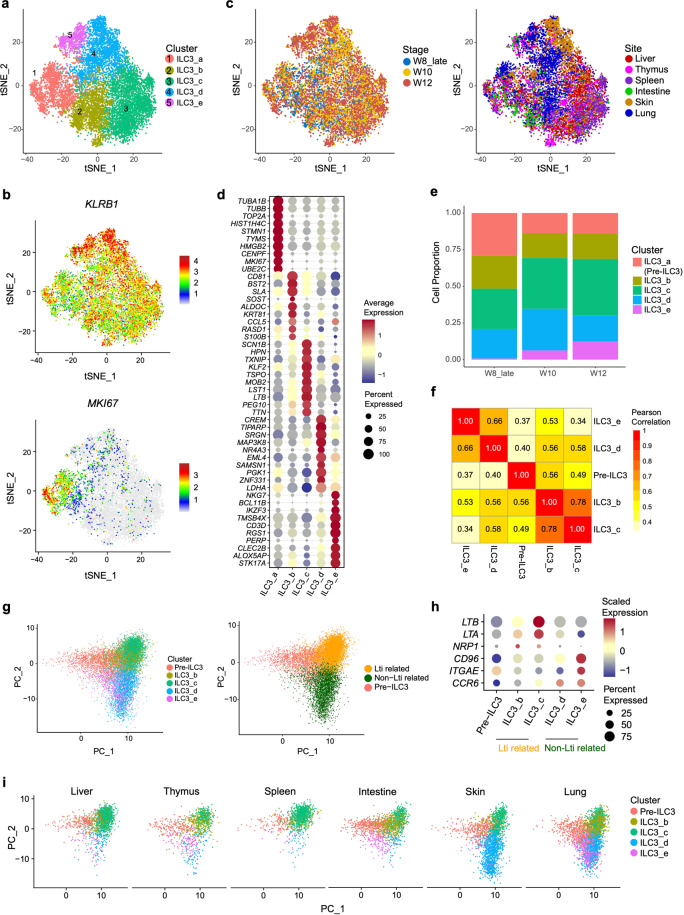
Data analysis reveals LTi-like and non-LTi development pathways from proliferating Pre-ILC3. **a***t*-SNE visualization of Group 3 ILCs with re-clustered subpopulations. Colors indicate cell subpopulations. **b** Expression of *KLRB1* and *MKI67* in ILC3 cells projected onto *t*-SNE. Colors indicate gene expression levels. **c** *t*-SNE visualization of ILC3 sub-clusters described in **a**, with stage (left panel) and site (right panel) information indicated by colors. **d** Dot plots show the expression level of top 10 DEGs in ILC3_a, ILC3_b, ILC3_c, ILC3_d, ILC3_e. Colors represent the average expression and size encodes the proportion of gene-expressing cells. **e** Bar graph shows the proportions of ILC3_a (Pre-ILC3), ILC3_b, ILC3_c, ILC3_d, ILC3_e in different development stages. Colors indicate different cell types. **f** Heatmap of pearson correlation matrix among five ILC3 sub-clusters. Colors and numbers indicate correlation coefficient. **g** PCA visualization of all ILC3 sub-clusters (left panel), which can be further clustered into Pre-ILC3, LTi-related and non-LTi groups based on PCA analysis (right panel). **h** Dot plots show the scaled expression level of indicated genes in ILC3 sub-clusters. Colors represent the scaled expression and size encodes the proportion of gene-expressing cells. **i** The tissue distributions of ILC3 sub-cluster were projected to PCA. Colors indicate different ILC3 sub-clusters.

In accordance with flow cytometric results, Group 3 ILC was the major ILC population with five subpopulations (Fig. 6a; Supplementary information, Table S5), although the expression of *NCR2* (encoding NKp44) in ILC3 cells was very low, consistent with the protein expression pattern (Supplementary information, Figs. S2c and S9a). ILC3_a was a proliferating subset, showing the higher expression of DNA replication-regulated genes (*TUBA1B*, *TUBB* and *TOP2A*) and *MKI67*, but lower expression of ILC3-related genes, such as *RORC* and *KIT*, than other subsets (Fig. 6b, c; Supplementary information, Fig. S9a), indicative of its precursor identity. Meanwhile, the dynamic change of ILC3_a proportion was similar to ILC1 precursor (Figs. 4i and 6e). On the other hand, the proportion of ILC3_e gradually increased along with development, indicating that this subset might represent the mature ILC3s, and higher expression of *KLRB1* in ILC3_e also suggested that they might be fully mature (Fig. 6b–e). Intriguingly, ILC3_e also highly expressed genes encoding NK and T cell surface markers and TFs, such as *NKG7*, *CD3D*, *IKZF3*^85^ and *BCL11B*, as seen in CCR9_ILC2s identified above (Fig. 6d). ILC3_b was featured by *KRT81* and *CCL5*. The LTi-featured genes *LTB* was expressed in both ILC3_b and ILC3_c cells (Fig. 6d).

To determine the correlation of these five ILC3 subsets, pearson correlation analysis was employed. The results showed two modules, one gathering ILC3_b and ILC3_c, and the other with ILC3_d and ILC3_e. ILC3_e showed the least similarity with ILC3_a compared with the other ILC3 subsets (Fig. 6f). This result promoted us to propose the divergent developmental pathways of LTi and non-LTi ILC3 as known in mouse. Group 3 ILC cells were re-grouped into three major sub-clusters, named Pre-ILC3, LTi and Non-LTi ILC3-related clusters, by components analysis (PCA) (Fig. 6g). Consistent with pearson correlation analysis results, Pre-ILC3 approximately overlaid with ILC3_a. ILC3_b and ILC3_c formed the LTi-related cluster, with increased expression of LTi characteristic genes such as *LTA* and *LTB*. *NRP1*, expressed by LTi-like group 3 ILCs in smokers’ lungs and associated with ectopic lymphoid aggregates,^86^ was also enriched in ILC3_b and ILC3_c (Fig. 6h). Importantly, ILC3_b and ILC3_c were preferably located in lymphoid tissues including thymus and spleen, as well as intestine where mLNs will be formed in the second trimester (Fig. 6i; Supplementary information, Fig. S9b), correlating the function of LTi to induce the formation of lymphoid tissues. As expected, ILC3_d and ILC3_e clustered together, forming the Non-LTi-related ILC3 developmental pathway, with expression of *CD96* and *ITGAE*,^11,87,88^ the genes to promote NKp44^+^ ILC3 adhesion to epithelial cells. Chemokine receptor-encoding gene *CCR6*, which has an important role in the localization of NKp44^+^ ILC3s,^11^ was also highly enriched in both ILC3_d and ILC3_e (Fig. 6h). In summary, our data illuminate the branched development pathways of LTi and non-LTi ILC3s from ILC3 precursors in early human early fetal stages, which is consistent with mouse studies.^89,90^

### TF network and dynamics of cell cycle state along with ILC development

**Fig. 7:**
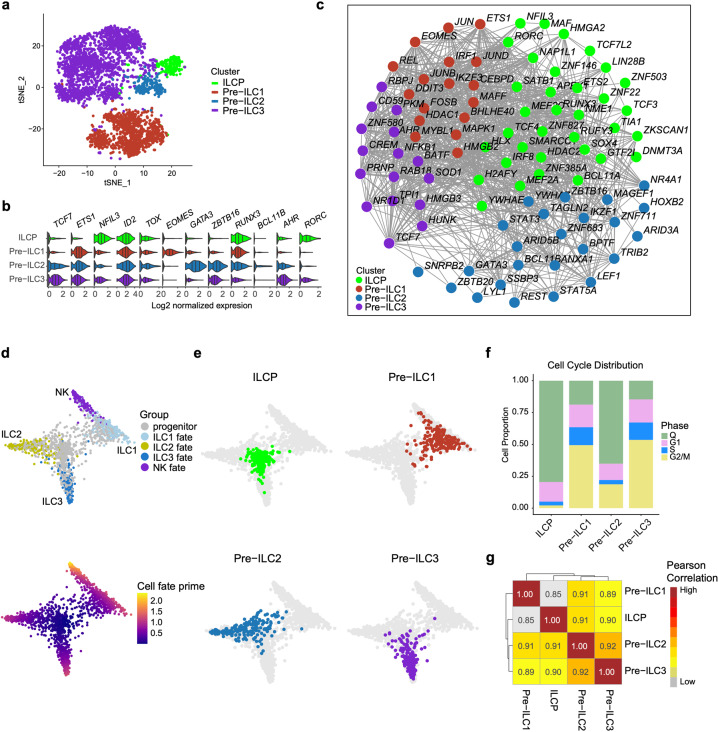
TF network and dynamics of cell cycle state along with ILC specialization. **a***t*-SNE visualization of ILCP from Fig. 2a, Pre-ILC1 from Fig. 4a, Pre-ILC2 from Fig. 5a and Pre-ILC3 from Fig. 6a. **b** Violin plots show the expression of featured TF-encoding genes in different cell clusters. **c** The correlation network of TFs of ILCP and three Pre-ILCs. All TFs in each cluster were used to construct network; nodes (TFs) with more than 3 edges are shown. Colors indicate cell types (**a**–**c**). **d** Fully annotated STEMNET map visualizes fate probabilities of progenitors toward mature NK, ILC1, ILC2 and ILC3 cells. Colors indicate cell types (upper panel) and the possibility of progenitor cells developing into the corresponding lineage (below panel). **e** Visualization of the probabilities of different progenitors and precursors to develop toward the specific lineages. Colors indicate cell types. **f** Proportions of the cells with different cell cycle statuses in each cluster are shown in bar graph. Colors represent cell cycle statues. **g** Heatmap of pearson correlation matrix among ILCP and three Pre-ILCs. Colors indicate correlation coefficient.

The annotation of ILCP in our study was based on the reported features of human ILC progenitors/precursors in two previous studies. The ILC progenitors identified in human tonsil with CD34^+^CD117^+^IL-1R1^+^ phenotype also expressed high level fo RORC. Meanwhile, ILCPs are identified as a subset of Lin^–^ CD45^+^CD7^+^CD127^+^CD117^+^ cells in human peripheral blood, adult and fetal tissues (Supplementary information, Fig. S10a).^21,36^ Three types of ILC lineage specific precursors were also annotated, predicted by their distinctive proliferating status together with low expression of respective ILC featured genes. To uncover the molecular basis operating ILC lineage commitment, we combined these ILC precursors with ILCP for further analysis. These four cell populations formed distinguished clusters based on their original identities, visualized by *t*-SNE (Fig. 7a; Supplementary information, Fig. S10b and Table S7). In coincidence with studies in mouse,^25,27^ ILCP had the upregulated expression of *NFIL3*, *ID2* and *TOX* (Fig. 7b), indicating that the multi-potent lymphoid progenitors had been specialized to the ILC lineages. The expression of ILC lineage-specific TF-coding genes, including *EOMES*, *GATA3*, and *AHR*, remained low at ILCP stage, and was subsequently upregulated in the Pre-ILC1, Pre-ILC2 and Pre-ILC3 cells, respectively (Fig. 7b). Notably, *TCF7*, encoding the important TF (TCF1) for T cell specialization both in human and mouse,^46,91^ was also highly expressed in murine early innate lymphoid progenitors (EILPs) with restricted ILC potential to generate both NK and helper ILCs.^19,92^ In addition, in *Tcf7*^*–/–*^ mice, Ly49a^+^ NK cells, ILC2s and immature ILC2s, were sharply reduced and ILC2-mediated type 2 immune responses to helminth infection was lacking,^93–95^ highlighting the importance of *TCF7* on ILC2 development and function. However, *TCF7* was barely expressed in ILCP but highly expressed in Pre-ILCs, especially Pre-ILC3 (Fig. 7b), suggesting that at human early fetal stages, *TCF7* might be involved in distinct helper ILC lineage restriction, but not essential for common ILC lineage commitment or development of killer ILCs. Expression of *ZBTB16* (encoding PLZF) discriminates ILC precursors without LTi potential from ID2 highly expressing common helper ILC progenitors (CHILP) in murine.^29,33^ *ZBTB16* expression level was low in ILCP but high in Pre-ILC2 and Pre-ILC3; meanwhile, *ZBTB16* expression was also low in Pre-ILC1 that could generate NK cells (Fig. 7b), indicating that *ZBTB16* may play an important role in LTi development at human early fetal stages. Additionally, *IRF8*, encoding the essential TF for DC development and reported to be expressed in subsets of ETPs,^46,59,69^ was also enriched in ILCP, suggesting the strong correlations among the development pathways of ILCs, T cells and DCs (Fig. 7c). Many T cell-related TFs were expressed in both ILCP and ILC precursors, including *BCL11A* and *TCF3* in ILCP, *LEF1*, *ETS1* and *IKZF3* in Pre-ILC1, *LYL1* and *BCL11B* in Pre-*ILC2* and *TCF7* in Pre-ILC3^61,96,97^ (Fig. 7c; Supplementary information, Fig. S10c), suggesting the involvement of similar transcription machinery for T cell and ILC development.^98^

Consistent with their annotations, ILCP showed less primed lineage potential than the three individual ILC precursors that were restricted to the respective ILC lineage branch, predicted by STEMNET. Notably, Pre-ILC1 cells had both ILC1 and NK cell potentials (Fig. 7d, e). Interestingly, cell cycle analysis showed that the majority of ILCPs were quiescent (Fig. 7f; Supplementary information, Table S6). In contrast, most of ILC precursors were in active cell cycle status, as mentioned above. The proportion of resting cells varied among three types of ILC precursors, and that in Pre-ILC1 and Pre-ILC3 was similar, while Pre-ILC2 showed the highest proportion. Pearson correlation analysis showed that ILCP was less similar to Pre-ILC1, which might be due to the NK precursors in ILC1_a cells (Fig. 7g). These data were supported by the early bifurcation of developmental pathways of cytotoxic ILCs and helper ILCs, which is conserved in mouse and human. In summary, we characterize the TFs that might govern the ILC lineage decision at ILCP to ILC precursor stage, accompanied by dynamic cell cycle status.

## Discussion

ILCs, the innate counterparts of T cells, are increasingly appreciated due to their irredundant role in host immune system. The developmental pathways of ILCs in mouse have been explored in recent years, however, the ontogeny of human ILCs is poorly understood. In this study, we investigated human ILC development at fetal stage by scRNA-seq and biological validation. A linear and successive developmental path from HSCs, to LP1, LP2, ILCP, and to each ILC-specific precursor was revealed by our data. Due to the limited samples, the precise progenitor–progeny relationship of these progenitor/precursors needs further investigation.

HSCs go through the sequential restriction of multi-lineage differentiation potential, resulting in lineage commitment during hematopoiesis, the process controlled by multiple-layer of regulatory mechanisms. In adult human bone marrow and cord blood, the lymphoid-primed progenitors still retain myeloid potential but not erythroid potential.^99–101^ These progenitors, named “lymphoid-primed multipotent progenitors” or “multilymphoid progenitors” (MLP), were identified as high expression of L-selection or CD34^+^CD38^–^Thy-1^–^CD45RA^+^.^100,101^ The mouse LMPPs are featured by CD135 expression compared with HSCs.^102,103^ Up-regulation of CD127 leads to the final commitment to lymphoid lineage and elimination of myeloid potential. T, B and ILC cells lineage fate might be decided thereafter. CD135^+^α4β7^-^HSA^int^ unbiased CLPs, the direct progeny of LMPPs, give rise to HSA^hi^ and HSA^lo^ CLPs which are B-biased and T/ILC biased, respectively.^104^ ILC lineage commitment is further concreted, and the up-regulation of α4β7 and loss of CD135 expression suggest the loss of B and T cell potential successively.^105^ CD127^+^CD117^+^ ILCPs discovered by Di Santo’s group have been proved to be HSC-derived through transplantation experiments,^36^ but the definitive intermediate stage that bridges HSCs and ILCPs was lacking. IL-3RA^+^CD127^+^CD34^+^ lymphoid progenitors identified at transcription and protein levels in this study might be the fetal human equivalents of LMPPs mentioned above, regarding their tri-lymphoid and myeloid potential predicted by STEMNET analysis as well as functional validation at bulk and single-cell levels. The IL-3RA^+^ lymphoid progenitors might be correlated with the first wave thymus-seeding progenitors with bipotent T/ILC potential as reported in the mouse.^106,107^ Diminished DC potential along ILC lineage commitment has been found in human tonsil and mouse bone marrow.^19,21^ Based on the increased expression of *IRF7* and *IRF8* together with the decreased expression of *IL7R* and *CD7* compared with LP1, the LP2 cluster might represent the intermediate stage between multi-potent lymphoid progenitors and ILC-committed progenitors. These cells might be equivalent to the specified early innate lymphoid progenitors (EILPs), possessing both ILC and DC potentials, named by Bhandoola’s group.^19^ However, the difference of lineage restrictions between LP1 and LP2 needs to be further investigated.

Single-cell clonal assay in the previous study using fetal liver Lin^–^CD45^+^CD7^+^CD127^+^CD117^+^ cells^36^ which contained ILCP and Pre-ILC3 identified in this study, showed their heterogeneous ILC differentiation potential in vitro. 56% and 39% clones from OP9 and OP9-DL4 coculture systems respectively, showed ILC3 uni-product, suggesting that the presence of ILCPs that have already committed to ILC3 lineage. Less than 10% of these cells had multi-lineage potential to generate all groups of ILCs suggested by both coculture systems. Potential markers to distinguish each ILC precursors have been provided, however, the ideal surface markers to perfectly differ Pre-ILC3 from ILCP are hardly defined. Due to the limited and precious samples, the precise progenitor-progeny relationship of these progenitor/precursors warrants further investigation. The fetal ILCPs also showed intestine-biased feature, consistent with the local-differentiation characteristics of ILCs. In addition to ILCPs, the precursors of individual ILC subsets were identified in our study, and several common features shared by these precursors were revealed. For instance, the expression level of the featured genes of each ILC subset was lower in the precursors than in mature ILCs, and more precursors were detected at 8 PCW than 10 and 12 PCW. More importantly, despite the potential heterogeneity of these cells, majority of the precursors were proliferative and expressed DNA replication-related genes. In contrast, the ILCPs and mature ILCs were quiescent. The similar dynamics of cell proliferation is also found in the T cell development that involves multiple runs of cell proliferation. In the initial steps of T cell development, the most primitive ETPs in thymus are quiescent, then the ETPs start to proliferate, simultaneously commit to T cell lineage.^46,59,62^ Given the similarity of T cell and ILC development, it was therefore likely that ILC precursors might proliferate when committed to individual ILC lineage, and the final step of ILC maturation might not involve cell expansion. Potential markers to distinguish each ILC precursors have been also provided, however, ideal surface marker to perfectly differ Pre-ILC3 from ILCP is to be identified and moreover, functional validation needs further investigation.

The heterogeneity and tissue distribution of individual ILC populations have been studied in the adult humans and mice, showing the varied phenotypes and functions of ILCs in different tissues. Our data also suggested the tissue-preferred distribution of ILC subsets with distinct molecular features. For example, *CXCR4* expressed in ILC1_b cells might be involved in the migration of these cells into skin. ILC_1d cells were heterogenous, containing ILC1-biased and NK-biased cells. Although the ILC1-biased and NK-biased cells could be distinguished by lineage-specific feature genes, their lineage bifurcation was not obvious in the trajectory analysis. They were possibly relatively immature at this early fetal stage which could develop into mature CD56^+^ ILC1 cells and NK cells, respectively.^1,43^ Moreover, *CCR9*-expressing ILC2 subset was mainly located in thymus, consistent with the function of CCR9-CCL25 in ETP settling in thymus. Notably, this subset of ILC2 was CRTH2^–^ and CD117^–^, suggestive of the unconventional ILC2s identity since these cells had the comparable GATA3 but low RORγt levels compared with the conventional ILC2s and ILC3s, respectively. Notably, the chemokine receptor CCR9 is also highly expressed on ILC2P (Lin^–^Sca-1^hi^ID2^hi^GATA3^hi^KLRG1^–^) and mature ILC2 in bone marrow and small intestine of adult mouse. Significant reduction of GATA3^hi^ ILCs, including ILC2 and their progenitors, is observed in CCR9-deficient mice, especially in the intestine, suggestive of an important role of CCR9 in the recruitment of ILC2P to intestine.^34^ Besides, CCR9 expression was also reported on mouse fetal mesentery ILC2 progenitors and mature ILC2.^108^ Another *CCR6-* and *KIT*-expressing ILC2 subset without expression of *CRTH2* was speculated to be the fetal counterpart of the recently reported c-Kit^hi^CCR6^+^ ILC2s in the adult, harboring ILC2-ILC3 plasticity.

In summary, we have generated a high-resolution single-cell transcriptomic dataset that yields detailed information about the development and heterogeneity of human ILCs and related progenitor/precursors at fetal stage, as well as their transcriptional landscape and immunophenotypes, especially for the novel ILC subsets.

## Materials and methods

### Human samples

Healthy human embryonic and fetal samples were obtained following the elective medical termination of pregnancy at the Academy of Military Medical Sciences (the Fifth Medical Center of the PLA General Hospital). All experiments were performed in accordance with protocols approved by the Ethics Committee of the Affiliated Hospital of Academy of Military Medical Sciences (ky-2020-8-19), and local and state ethical guidelines and principles. The written informed consent was obtained before sample collection. The integrity of the samples has been confirmed and examined at multiple steps. From the beginning, the Obstetrics and Gynecology clinicians confirmed the collected embryos and fetus that were free of any known genetic or developmental abnormality. Before sample processing, the morphological examinations were performed to exclude samples with any potential developmental defects. Moreover, flow-cytometry analysis was used to examine whether the cellular profile of collected tissue was consistent with literature. Samples were classified according to the standard crown-rump length (CRL) measurement and were determined.^109,110^ Samples used in this study were from 8 to 12 PCW, with weeks indicating weeks post-fertilization. The gender of samples used for scRNA-seq was determined based on the expression of XIST and RPS4Y1 latterly. All the information of sample was summarized in Supplementary information, Fig. S1.

### Cell lines

OP9-DL4 (OP9 stromal line engineered to express human Delta-like-4 ligand) (Mus musculus, a generous gift from Dr. Nengming Xiao, Xiamen University) were maintained at 37 °C with 5% CO_2_ in α-MEM (GIBCO, 12561-056) supplemented with 20% Fetal Bovine Serum (FBS) (HyClone, SH30070.03), penicillin-streptomycin (GIBCO, 15140-122), 50 μM 2-mercaptoethanol (GIBCO, 21985-023) and l-glutamine.

### Flow cytometry

Cells were stained by the following antibodies: BV421 anti-human CD45 (BD, Cat#563879), APC-Cy7 anti-human CD3 (Biolegend, Cat#300426), APC-Cy7 anti-human CD5 (BD Cat#563516), APC-Cy7 anti-human CD11c (Biolegend, Cat#337218), APC-Cy7 anti-human CD14 (Biolegend, Cat#325620), APC-Cy7 anti-human CD19 (BD, Cat#560177), APC-Cy7 anti-human FcεRIα, (Biolegend, Cat#334632), APC anti-human ITGB7 (Biolegend, Cat#321207), APC anti-human CCR7 (Biolegend, Cat#353213), APC anti-human CCR9 (Biolegend, Cat#358907),BV605 anti-human CD117 (Invitrogen, Cat#313218), FITC anti-human CD34 (BD, Cat#555821), PE-Cy7 anti-human CD127 (eBioscience Cat#25-1278-41), Alexa Fluor 647 anti-human CRTH2 (BD, Cat#558042), FITC anti-human CRTH2 (Biolegend, Cat#350108), FITC anti-human CD3 (Biolegend, Cat#300406), FITC anti-human CD5 (Biolegend, Cat#300606), FITC anti-human CD14 (Biolegend, Cat#301804), FITC anti-human CD19 (Biolegend, Cat#302256), FITC anti-human CD11c (BD, Cat#561355), FITC anti-human FcεRIα (Biolegend, Cat#334640), PE anti-human CD161 (Biolegend, Cat#339903), APC-Cy7 anti-human CD45 (BD, Cat#557833), Percp anti-human CD56 (Biolegend, Cat#362525), PE-Cy7 anti-human IFN-g (BD, Cat#502528), BV421 anti-human IL13 (BD, Cat#563580), APC anti-human IL17a (BD, Cat#512333), BV510 anti-human CD19 (BD, Cat#562947), APC-Cy7 anti-human CD56 (Biolegend, Cat#362512), APC anti-human CD33 (Biolegend, Cat#366606), PE anti-human CD4 (Biolegend, Cat#300507), PE-Cy7 anti-human CD8 (BD, Cat#557746), BV510 anti-human CD7 (BD, Cat#563650), TotalSeq™-B0251 anti-human Hashtag 1 Antibody (Biolegend, Cat#394631), TotalSeq™-B0252 anti-human Hashtag 2 Antibody (Biolegend, Cat#394633), TotalSeq™-B0253 anti-human Hashtag 3 Antibody (Biolegend, Cat#394635), TotalSeq™-B0254 anti-human Hashtag 4 Antibody (Biolegend, Cat#394637), TotalSeq™-B0255 anti-human Hashtag 5 Antibody (Biolegend, Cat#394639), TotalSeq™-B0256 anti-human Hashtag 6 Antibody (Biolegend,Cat#394641)

### Preparation of single-cell suspensions

Fetal tissues including lung, intestine, skin, spleen and thymus were isolated and transferred to RPMI1640 medium (Gibco, 11875093) containing 10% FBS (HyClone, SH30070.03) on ice. Then, the tissues were washed with PBS for 3 times and transferred to pre-warmed digestion medium containing 100 μg/mL DNase I (Sigma-Aldrich, DN25) and 0.1 g/mL Collagenase I (Sigma, C2674) in PBS containing 10% FBS. The amount of enzyme depended on the size of tissues, such as 1–2 mL enzyme mixture for week 8 samples and 4–6 mL enzyme mixture for week 10 to 12 samples. Tissues were shaken vigorously for 30 s and further incubated at 37 °C for 30–40 min in incubator with general shaking every 6 min to release cells. Released cells passed through a 70 μm cell strainer (BD, 352350) and were collected in 15 mL tubes containing 8 mL FACS-buffer to neutralize enzymes. Cells were then collected by spinning at 500× *g* for 5 min, and suspended in FACS-buffer (1× PBS with 1% BSA) for subsequent staining.

Fetal liver was mechanically dissociated into single-cell suspension using scalpels and syringes. Cells were then filtered through a 70 μm cell strainer (BD, 352350) and collected by spinning at 400× *g* for 6 min. Resuspend in RBC lysis buffer (BD, 555899) for about 8 min to remove erythrocytes. After neutralization, remaining cells were collected in FACS buffer (1× PBS with 1% BSA) for subsequent staining.

### FACS and cell hashing for scRNA-seq

1–2 million cells from each tissue were resuspended in 50 µL Cell Staining Buffer (Biolegend, 420201) and 5 µL of Human TruStain FcX™ Fc Blocking reagent (Biolegend, 422301) was added and incubate for 10 min at 4 °C. Antibody pool of each hashtag antibody with titrated doseage (0.2 μL for 1 million cells), and sorting antibodies were prepared and added to the cell suspension, and incubated for 30 min at 4 °C. After washing, staining with 7-AAD was performed for about 5 min. Cells were sorted with gating strategy shown in Supplementary information, Fig. S1. on BD FACS Aria II and harvested in 200 μL PCR tube. Cells from each tissue were pooled into one 1.5 mL centrifuge tube. Cells were collected at 400× *g* at 4 °C for 5 min, resuspend in 50 uL FACS-buffer (1× PBS with 1 % BSA) to load into one lane of a 10× Chromium V3 chip. The cDNA preparation was performed following the instruction manual, and the hashtag library was prepared following the BioLegend TotalseqB guide. The final cDNA library and tag library were both sequenced on NovaSeq 6000. Cells were sequenced to an average depth of 0.1 M per cell for cDNA and 0.01 M per cell for hashtags.

### Coculture for assessment of B, T, NK and myeloid lineage potential at bulk and single-cell levels

Co-cultivation on the OP9 stromal line engineered to express human Delta-like-4 ligand (OP9-DL4, a generous gift from Dr. Nengming Xiao, Xiamen University) and OP9 stromal cells with SCF, FL, IL-7 and IL-15 was used to test the B, T, NK and myeloid lineage potential of Lin^–^CD45^+^CD127^+^CD34^+^IL-3RA^+/–^ progenitors. For bulk culture, 1–1.5 × 10^4^ stromal cells were pre-seeded in 24-well round bottom plates one night before culture. Freshly FACS-sorted progenitors (50–200 cells) were seeded onto established, nonirradiated OP9-DL4 or OP9 stromal cells. For single-cell culture, 3–5 × 10^3^ stromal cells were pre-seeded in 96-well plates one night before culture. Lin^–^CD45^+^CD127^+^CD34^+^IL-3RA^+^ progenitors were sorted in single-cell mode and then plated on the stromal cells. Cells were cultured in α-MEM (GIBCO, 12561-056) containing 20% Fetal Bovine Serum (HyClone, SH30070.03), 50 μM 2-mercaptoethanol (GIBCO, 21985-023), penicillin-streptomycin (GIBCO, 15140-122), L-glutamine in the presence of SCF (5 ng/mL), IL-7 (5 ng/mL), Flt3 ligand (5 ng/mL) and IL-15 (10 ng/mL) (cytokines from PeproTECH) for 8–14 days. Stromal cells were passaged when the confluence reached about 80%–95%. Co-cultured cells were separated by 0.25% Trypsin-EDTA (Sigma, 59428 C) solution and collected by spinning at 500× *g* for 5 min at 4 °C. All cultures were analyzed in parallel with negative control wells that contained OP9-DL4 or OP9 stromal cells and identical culture medium without progenitors. T, B, NK and myeloid cells were identified as positive for its respective lineage markers and negative for other lineage markers. For clonal assay, clones were required to have over ten CD45^+^ cell-gated events (of the appropriate cell-surface phenotype) to be considered positive.

### Coculture for assessment of ILC lineage potential at bulk and single-cell levels

For bulk culture, 1–1.5 × 10^4^ OP9-DL4 stromal cells were pre-seeded in 24-well round bottom plates one night before co-culture. Freshly FACS-sorted Lin^–^CD45^+^CD127^+^CD34^+^IL-3RA^+/–^progenitors (150 cells) or ILCP (Lin^–^CD45^+^CD7^+^CD127^+^CD117^+^) were seeded into the wells with pre-seeded OP9-DL4 stromal cells. For single-cell culture, 3–5 × 10^3^ OP9-DL4 stromal cells were pre-seeded in 96-well round bottom plates one night before co-culture. Cells of Lin^–^CD45^+^CD127^+^CD34^+^IL-3RA^+^ progenitors were sorted in single-cell mode and then plated on the stromal cells. Cells were cultured in Yssel’s medium supplemented with 2% human AB serum (GeminiBio, 100–512) as Dr. James P. Di Santo’s group described in the presence of IL-2, IL-7, IL-1β, IL-23, IL-25 and IL-33 (PeproTECH) for 10–14 days.^36^ Stromal cells were passaged when the confluence reached about 80%–95%. Co-cultured cells were treated by 0.25% Trypsin-EDTA (Sigma, 59428 C) solution and collected by spinning at 500× *g* for 5 min at 4 °C. All cultures were analyzed in parallel with negative control wells that contained OP9-DL4 stromal and identical culture medium without progenitors. To detect the cytokine expression, cells were stimulated with cell stimulation cocktail (Tonbo, TNB-4975), 2 μL per sample, for 4 h. Intracellular staining of cytokines was performed with Cytofix/Cytoperm Kit (BD, 554714).

### Yssel’s medium

Yssel’s medium was prepared in house by using IMDM (Invitrogen, 31980-030) plus 0.25% (w/v) BSA (Sigma, A9647), 1.8 mg/L 2-amino ethanol, 40 mg/L Apo-transferrin, 5 mg/L insulin and Penicillin/Streptomycin.

### Sequencing and pre-processing data

Single-cell RNA-seq library was prepared and performed on the Chromium platform (10× Genomics), using the single cell expression 3′ profiling chemistry combined with cell hashing. Cell-hashing antibody (HTO)-labeled cells from 2–3 tissues were pooled together and washed with RPMI-1640 immediately before loading on the 10× controller. Then the library was constructed following the manufacturer’s protocol, with some additional steps for the amplification of HTO barcodes. The raw data were aligned to the hg19 reference genome using cellranger count (Version 3.0), and the hashed cells were demultiplexed using HTODemux function in Seurat 3, the cells aligned to doublet and negative group were removed from further analysis.

Single-cell data analysis (including quality control, data normalization, dimension reduction, clusters detection) were performed using Seurat 3 in R. Firstly, count matrices of gene expression derived from the cellranger output were subjected to Seurat. Low quality cells (less than 1000 genes, less than 1000 UMIs or less than 20 percent UMIs mapping on mitochondria in one cell) were filtered before data normalization was performed. The filted data were integrated using method based on identification of ‘anchors’ between pairs of datasets following the tutorial at https://satijalab.org/seurat/v3.1/integration.html. Finally, PCA (principal component analysis) was performed and significant PCs were selected on the basis of the elbow of standard deviations of PCs. Next, UMAP, *t*-SNE, trimap, clustering analysis and re-clustering analysis were complemented using selected PCs using Seurat 3^111^ and Scanpy.^112^

### PAGA analysis

We quantified the connectivity of cell clusters using the PAGA method,^113^ using single-cell analysis package Scanpy based on significant PCs.

### SCENIC analysis

The transcriptional and regulatory characteristics of the different progenitors including HSPC, LP1, LP2, ILCP, ETP, pre pro-B populations, gene regulatory network analysis was performed using SCENIC.^114^ First, regulatory modules (regulons) were inferred from co-expression and DNA motif analysis. These regulons were then evaluated in each cell to ascertain their activity before a binary matrix was obtained. To profile the gene regulatory module features of all progenitors, the Spearman correlation coefficient between regulons was calculated, and only regulons with a correlation coefficient larger than 0.3 with at least one other regulon and activated in at least 30% cells in any progenitor clusters were included for visualization.

### STEMNET analysis

In order to infer the developmental potential of progenitor populations, STEMNET analysis was implemented.^115^ Firstly, we defined the most mature cells, such as B-cells, T-cells, ILCs, monocytes, macrophages, erythrocytes, megakaryocytes, and mast cells in our data as mature subsets (given that pre-pDCs in our data were immature, the mature pDCs in bone marrow^69^ were used as pDC subsets), allowing the STEMNET algorithm to calculate posterior probabilities of all the progenitor populations based on the highly expressed genes with average expression > 0.1.

### Gene expression network construction

To construct TFs correlation network during ILC specification, the differently expressed TF-encoding genes of each cluster were calculated and correlations between TFs were obtained using Pearson correlation. Then, only TFs with positive correlation with more than 3 other genes were retained for visualization using Cytoscape (https://cytoscape.org/).

### DEGs detection and cluster biomarker identification

DEGs were identified by running the ‘FindAllMarkers’ function in Seurat using the ‘Wilcox test’. All DEGs of specific clusters were listed in supplementary tables. Surface markers and transcription factors list were downloaded from Cell Surface Protein Atlas (http://wlab.ethz.ch/cspa/) and HumanTFDB3.0 (http://bioinfo.life.hust.edu.cn/HumanTFDB/).

### Cell cycle analysis

For cell cycle analysis, cell cycle-related genes consisting of a previously defined core set of 43 G1/S genes and 54 G2/M genes were used, which were listed in Supplementary information, Table S6. We used a way similar to that reported in Embryonic endothelial evolution towards first hematopoietic stem cells revealed by single-cell transcriptomic and functional analyses to classify the cycling phases of the cells.^116^ We calculated the average expression of each gene set as corresponding scores, and manually assigned cells to approximate cell cycle phases based on the scores.

### Trajectory analysis

We performed cellular trajectory reconstruction analysis using CytoTRACE Kernel of CellRank.^117^ Firstly, the KNN graph containing information about the undirected connectivities among cells was constructed. Then, a pseudotemporal ordering of cells was calculated using CytoTRACE,^118^ an algorithm that predicts cellular plasticity states based on the transcriptional diversity. Lastly, a transition matrix was constructed based on the KNN graph and pseudotemporal ordering and projected onto umap plot.

## Supplementary information

Supplementary information, Fig. S1

Supplementary information, Fig. S2

Supplementary information, Fig. S3

Supplementary information, Fig. S4

Supplementary information, Fig. S5

Supplementary information, Fig. S6

Supplementary information, Fig. S7

Supplementary information, Fig. S8

Supplementary information, Fig. S9

Supplementary information, Fig. S10

Supplementary information, Table. S1

Supplementary information, Table. S2

Supplementary information, Table. S3

Supplementary information, Table. S4

Supplementary information, Table. S5

Supplementary information, Table. S6

Supplementary information, Table. S7

## Data Availability

Further information and requests for resources and reagents should be directed to and will be fulfilled by the Lead Contact Dr. Bing Liu (bingliu17@yahoo.com). The scRNA-seq data of our study have been deposited at GEO (NCBI) with accession code GSE163587. This study did not generate new unique reagents.
