# Supplementary material for: Delineating spatiotemporal and hierarchical development of human fetal innate lymphoid cells
Source: Cell Res. 2021 Jul 8;31(10):1106–22. doi: 10.1038/s41422-021-00529-2 (PMC8486758; doi:10.1038/s41422-021-00529-2)
Supplement: Supplementary file 1 — Supplementary information, Fig. S1 [file 41422_2021_529_MOESM1_ESM.pdf]

Figure S1

Sample	Stage	Gender	Tissue	*Cell hashing with single-cell RNA sequencing 10x Genomics* Gating strategy	No. Hashtag (Total seqB)
F1	8 PCW	Male	Lung	P1: Lin$^-$CD45$^+$CD127$^+$CD34$^-$(ILC, 70%)	1
			Intestine	P2: Lin$^-$CD45$^+$CD34$^+$CD127$^+$(Lymphoid progenitor,10%)	2
			Liver	P3: Lin$^-$CD45$^+$CD34$^+$CD127$^-$(Other HSPC, 10%)	3
			Skin	P4: Lin$^-$CD45$^+$CD127$^-$CD34$^-$(Other lin, 10%)	4
F2	8 PCW	Female	Lung	P1: Lin$^-$CD45$^+$CD127$^+$CD161$^+$CD34$^-$(helper ILC, 75%)	1
			Intestine	P2: Lin$^-$CD45$^+$CD127$^-$CD161$^+$CD34$^-$(5%)	2
			Liver	P3: Lin$^-$CD45$^+$CD34$^+$CD127$^+$(Lymphoid progenitor,10%)	3
			Skin	P4: Lin$^-$CD45$^+$CD34$^+$CD127$^-$(Other HSPC, 5%)	4
			Spleen	P5: Lin$^-$CD45$^+$CD127$^-$CD34$^-$(Other lin, 5%)	5
			Thymus		6
F3	10 PCW	Female	Lung	P1: Lin$^-$CD45$^+$CD127$^+$CD161$^+$CD34$^-$(helper ILC, 75%)	1
			Intestine	P2: Lin$^-$CD45$^+$CD127$^-$CD161$^+$CD34$^-$(5%)	2
			Liver	P3: Lin$^-$CD45$^+$CD34$^+$CD127$^+$(Lymphoid progenitor,10%)	3
			Skin	P4: Lin$^-$CD45$^+$CD34$^+$CD127$^-$(Other HSPC, 5%)	4
			Thymus	P5: Lin$^-$CD45$^+$CD127$^-$CD34$^-$(Other lin, 5%)	6
F4	10 PCW	Male	Lung	P1: Lin$^-$CD45$^+$CD127$^+$CD34$^-$(ILC, 70%)	1
			Intestine	P2: Lin$^-$CD45$^+$CD34$^+$CD127$^+$(Lymphoid progenitor,10%)	2
			Liver	P3: Lin$^-$CD45$^+$CD34$^+$CD127$^-$(Other HSPC, 10%)	4
			Skin	P4: Lin$^-$CD45$^+$CD127$^-$CD34$^-$(Other lin, 10%)	5
			Thymus		6
F5	12 PCW	Male	Lung	P1: Lin$^-$CD45$^+$CD127$^+$CD161$^+$CD34$^-$(helper ILC, 75%)	1
			Intestine	P2: Lin$^-$CD45$^+$CD127$^-$CD161$^+$CD34$^-$(5%)	2
			Liver	P3: Lin$^-$CD45$^+$CD34$^+$CD127$^+$(Lymphoid progenitor,10%)	3
			Skin	P4: Lin$^-$CD45$^+$CD34$^+$CD127$^-$(Other HSPC, 5%)	4
			Spleen	P5: Lin$^-$CD45$^+$CD127$^-$CD34$^-$(Other lin, 5%)	5
			Thymus		6
F6	12 PCW	Female	Lung	P1: Lin$^-$CD45$^+$CD127$^+$CD161$^+$CD34$^-$(helper ILC, 75%)	1
			Intestine	P2: Lin$^-$CD45$^+$CD127$^-$CD161$^+$CD34$^-$(5%)	2
			Liver	P3: Lin$^-$CD45$^+$CD34$^+$CD127$^+$(Lymphoid progenitor,10%)	3
			Skin	P4: Lin$^-$CD45$^+$CD34$^+$CD127$^-$(Other HSPC, 5%)	4
			Spleen	P5: Lin$^-$CD45$^+$CD127$^-$CD34$^-$(Other lin, 5%)	5
			Thymus		6

Supplementary Figure 1 (related to Figure 1) The summarized information of samples collected in this study, including the developmental stage, gender, tissue, sorting strategy and hashtag number.

Human hematopoietic (liver), lymphoid (thymus; spleen) and non-lymphoid (intestine; skin; lung) tissues were collected from the fetal samples at indicated developmental stage, subjected to digestion and processed to single cell solution (described in Methods). Cells were labeled with hashtag oligos referring to different tissues of one sample and sorted based on the sorting strategy in Figure S1 for 10x Genomics sequencing.
