# Supplementary material for: Delineating spatiotemporal and hierarchical development of human fetal innate lymphoid cells
Source: Cell Res. 2021 Jul 8;31(10):1106–22. doi: 10.1038/s41422-021-00529-2 (PMC8486758; doi:10.1038/s41422-021-00529-2)
Supplement: Supplementary file 2 — Supplementary information, Fig. S2 [file 41422_2021_529_MOESM2_ESM.pdf]

Figure S2

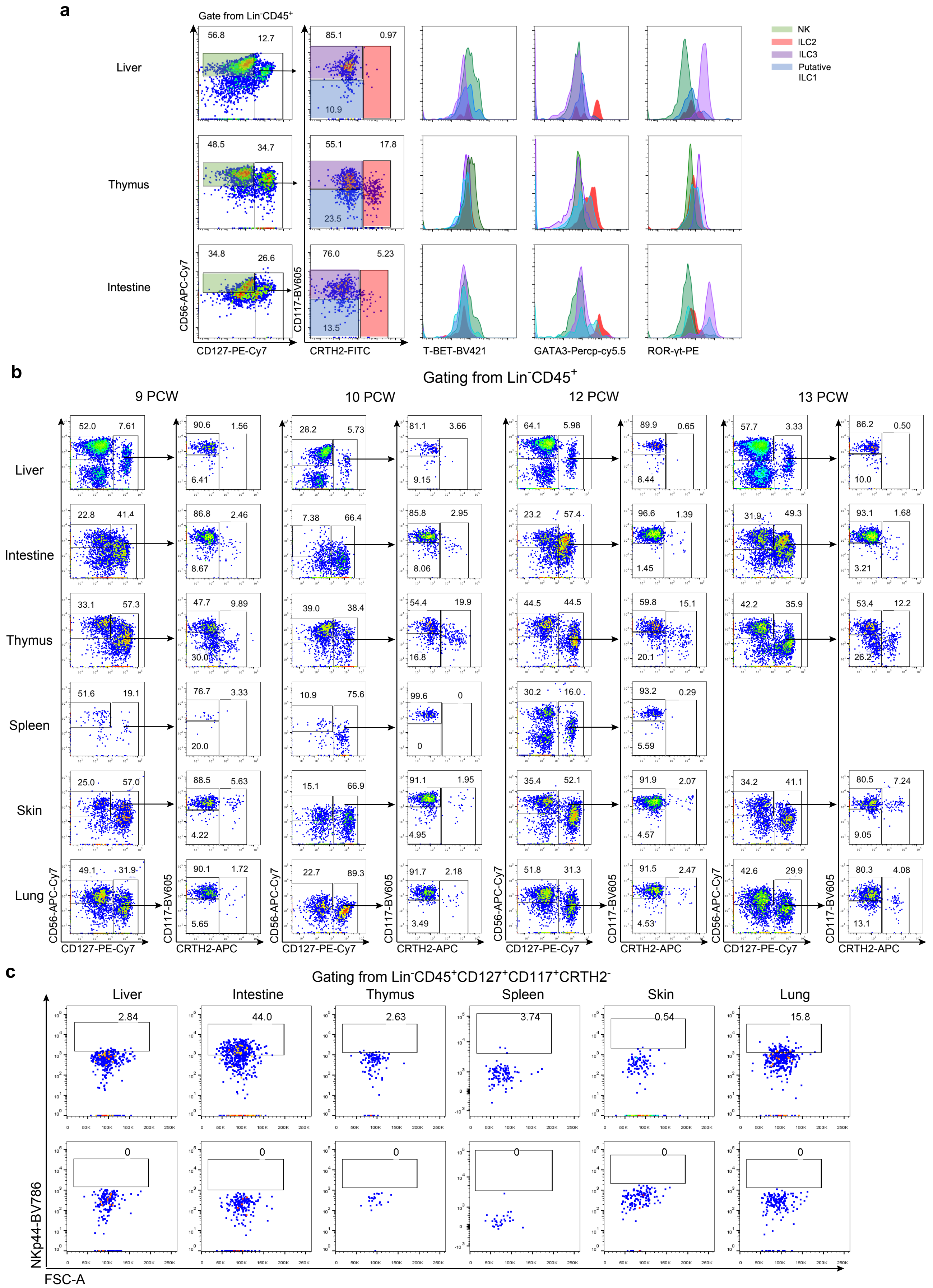
a
Gate from Lin-CD45+
Liver
Thymus
Intestine
56.8
12.7
85.1
0.97
10.9
48.5
34.7
55.1
17.8
23.5
34.8
26.6
76.0
5.23
13.5
CD56-APC-Cy7
CD117-BV605
CD127-PE-Cy7
CRTH2-FITC
T-BET-BV421
GATA3-Percp-cy5.5
ROR-γt-PE
NK
ILC2
ILC3
Putative ILC1
b
Gating from Lin-CD45+
9 PCW
10 PCW
12 PCW
13 PCW
Liver
Intestine
Thymus
Spleen
Skin
Lung
CD56-APC-Cy7
CD127-PE-Cy7
CD117-BV605
CRTH2-APC
c
Gating from Lin-CD45+CD127+CD117+CRTH2-
Liver
Intestine
Thymus
Spleen
Skin
Lung
2.84
44.0
2.63
3.74
0.54
15.8
0
0
0
0
0
0
NKp44-BV786
FSC-A

Supplementary Figure 2 (related to Figure 1) The tissue distribution of mature ILCs in different tissues at different developmental stages is shown by flow cytometric analysis.

(a) Expression of intracellular transcription factors (T-BET, GATA-3, and RORγt) in NK, ILC2, ILC3 and putative ILC1 in different tissues. **(b)** Representative flow cytometry results of killer ILCs and helper ILCs subpopulations in different tissues at each stage. **(c)** Expression of NKp44 were detected in $CRTH2^{-}CD117^{+}$ ILC3. Flow cytometric analysis of NKp44 protein level of $CRTH2^{-}CD117^{+}$ ILC3 from each tissue (upper panel) and corresponding fluorescence minus one controls (below panel).
