# Supplementary material for: Delineating spatiotemporal and hierarchical development of human fetal innate lymphoid cells
Source: Cell Res. 2021 Jul 8;31(10):1106–22. doi: 10.1038/s41422-021-00529-2 (PMC8486758; doi:10.1038/s41422-021-00529-2)
Supplement: Supplementary file 3 — Supplementary information, Fig. S3 [file 41422_2021_529_MOESM3_ESM.pdf]

Figure S3

a

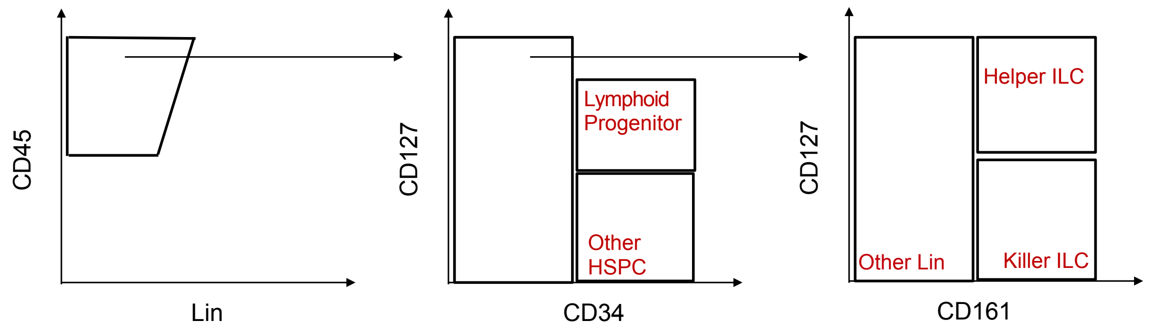

Gate from 7-AAD-
CD45
Lin
CD127
CD34
Lymphoid Progenitor
Other HSPC
CD127
CD161
Helper ILC
Killer ILC
Other Lin

b

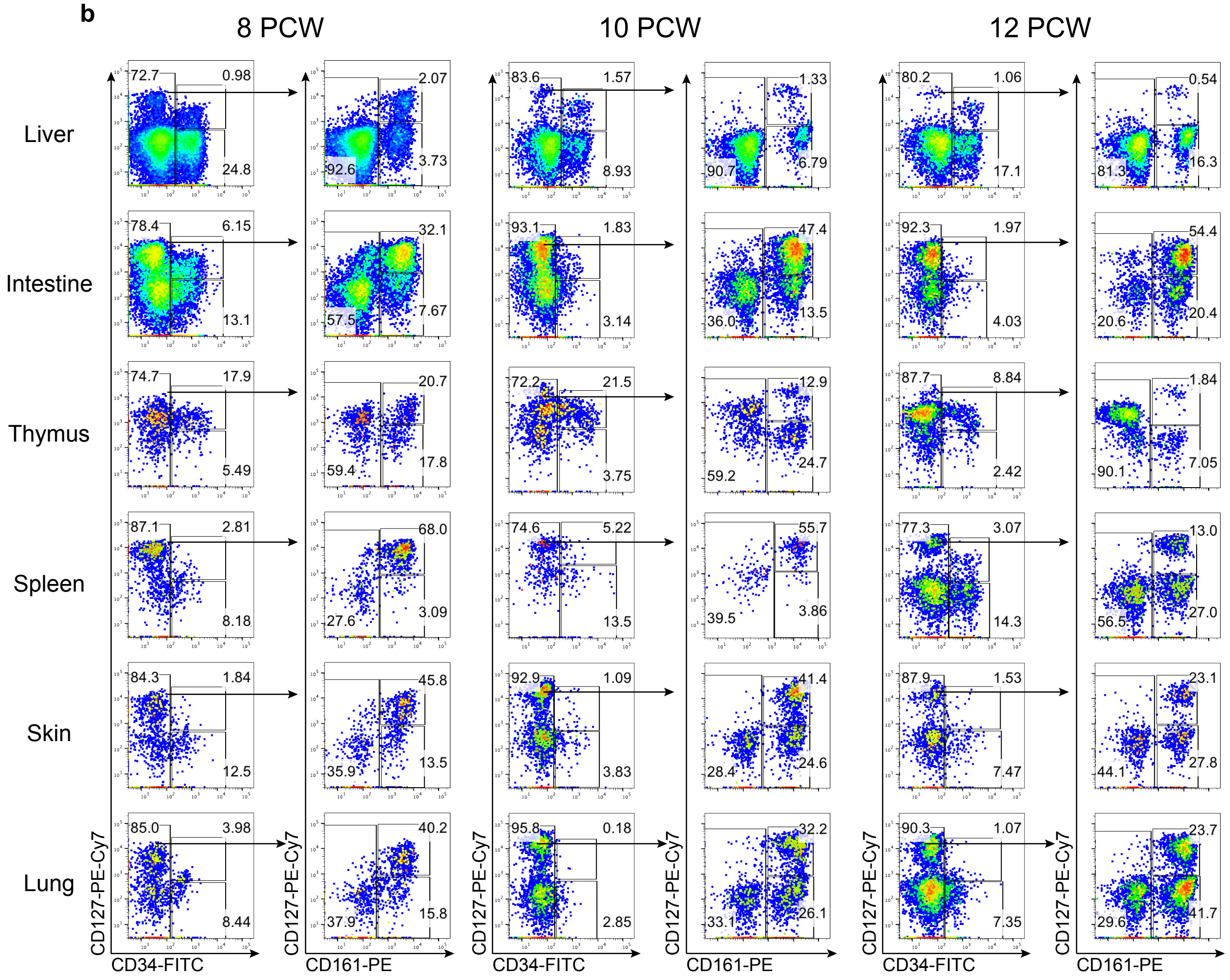

8 PCW
10 PCW
12 PCW
Liver
Intestine
Thymus
Spleen
Skin
Lung
CD127-PE-Cy7
CD34-FITC
CD161-PE
72.7
0.98
24.8
2.07
92.6
3.73
83.6
1.57
8.93
1.33
90.7
6.79
80.2
1.06
17.1
0.54
81.3
16.3
78.4
6.15
13.1
32.1
57.5
7.67
93.1
1.83
3.14
47.4
36.0
13.5
92.3
1.97
4.03
54.4
20.6
20.4
74.7
17.9
5.49
20.7
59.4
17.8
72.2
21.5
3.75
12.9
59.2
24.7
87.7
8.84
2.42
1.84
90.1
7.05
87.1
2.81
8.18
68.0
27.6
3.09
74.6
5.22
13.5
55.7
39.5
3.86
77.3
3.07
14.3
13.0
56.5
27.0
84.3
1.84
12.5
45.8
35.9
13.5
92.9
1.09
3.83
41.4
28.4
24.6
87.9
1.53
7.47
23.1
44.1
27.8
85.0
3.98
8.44
40.2
37.9
15.8
95.8
0.18
2.85
32.2
33.1
26.1
90.3
1.07
7.35
23.7
29.6
41.7

Supplementary Figure 3 (related to Figure 1) Flow cytometric analysis of samples used for 10x genomics sequencing

(a) Sorting strategy of human fetal ILC associated populations, which were all gated from 7-AAD$^-$Lin$^-$CD45$^+$, CD34$^+$CD127$^+$ lymphoid progenitors, CD34$^+$CD127$^-$HSPCs, CD34$^-$CD161$^+$CD127$^{+/-}$ ILCs and CD34$^-$CD161$^-$ cells were sorted simultaneously and mixed at certain ratio described in Figure S1 from each tissue. **(b)** Representative flow cytometry plots show frequencies of five cell populations above from an individual sample at each stage.
