# Supplementary material for: Delineating spatiotemporal and hierarchical development of human fetal innate lymphoid cells
Source: Cell Res. 2021 Jul 8;31(10):1106–22. doi: 10.1038/s41422-021-00529-2 (PMC8486758; doi:10.1038/s41422-021-00529-2)
Supplement: Supplementary file 4 — Supplementary information, Fig. S4 [file 41422_2021_529_MOESM4_ESM.pdf]

Figure S4

a

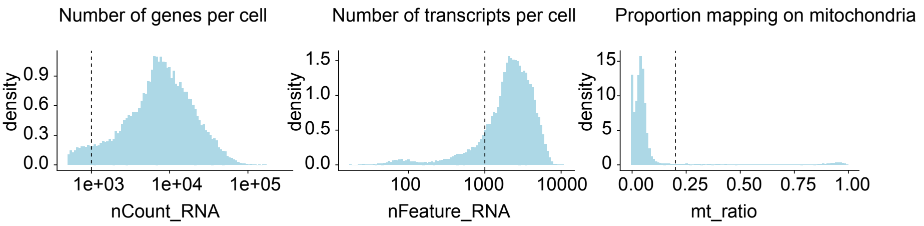

b

	Liver	Thymus	Spleen	Intestine	Skin	Lung	Total
W8_late	2769	255	277	3058	1616	1536	9511
W10	3461	2096	NA	1375	2075	2208	11215
W12	3247	1869	1835	672	1177	1707	10507
Total	9477	4220	2112	5105	4868	5451	31233

c

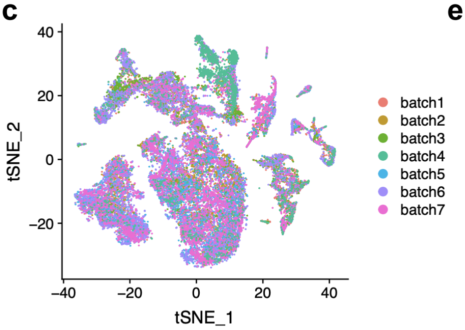

e

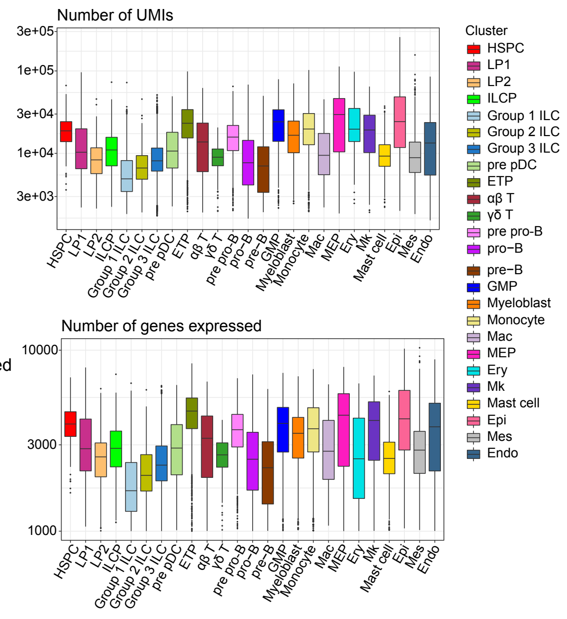

d

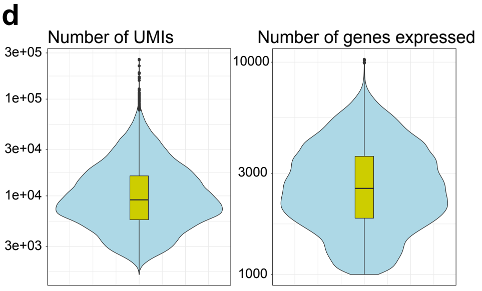

Supplementary Figure 4 (related to Figure 2) Technical information and quality control of scRNA-seq library

(a) Quality control for 10X Genomics data by genes (left panel), transcripts (middle panel) and ratio of mitochondria gene (right panel). The threshold for final analysis was set at gene number >1,000 per cell and ratio of mitochondria gene <0.2. **(b)** Numbers, tissue and stage information of cells used in final analysis. **(c)** tSNE showing minimal batch effect among single cell libraries. **(d)** Violin plots of average UMI and gene numbers of scRNA-seq data. For box plot within each violin plot, center black lines indicate median values, boxes range from 25th to 75th percentiles, and whiskers correspond to 1.5XIQR. **(e)** UMI (upper panel) and gene numbers (below panel) of identified cell clusters. For box plot within each violin plot, center black lines indicate median values, boxes range from 25th to 75th percentiles, and whiskers correspond to 1.5XIQR.
