# Supplementary material for: Delineating spatiotemporal and hierarchical development of human fetal innate lymphoid cells
Source: Cell Res. 2021 Jul 8;31(10):1106–22. doi: 10.1038/s41422-021-00529-2 (PMC8486758; doi:10.1038/s41422-021-00529-2)
Supplement: Supplementary file 5 — Supplementary information, Fig. S5 [file 41422_2021_529_MOESM5_ESM.pdf]

Figure S5

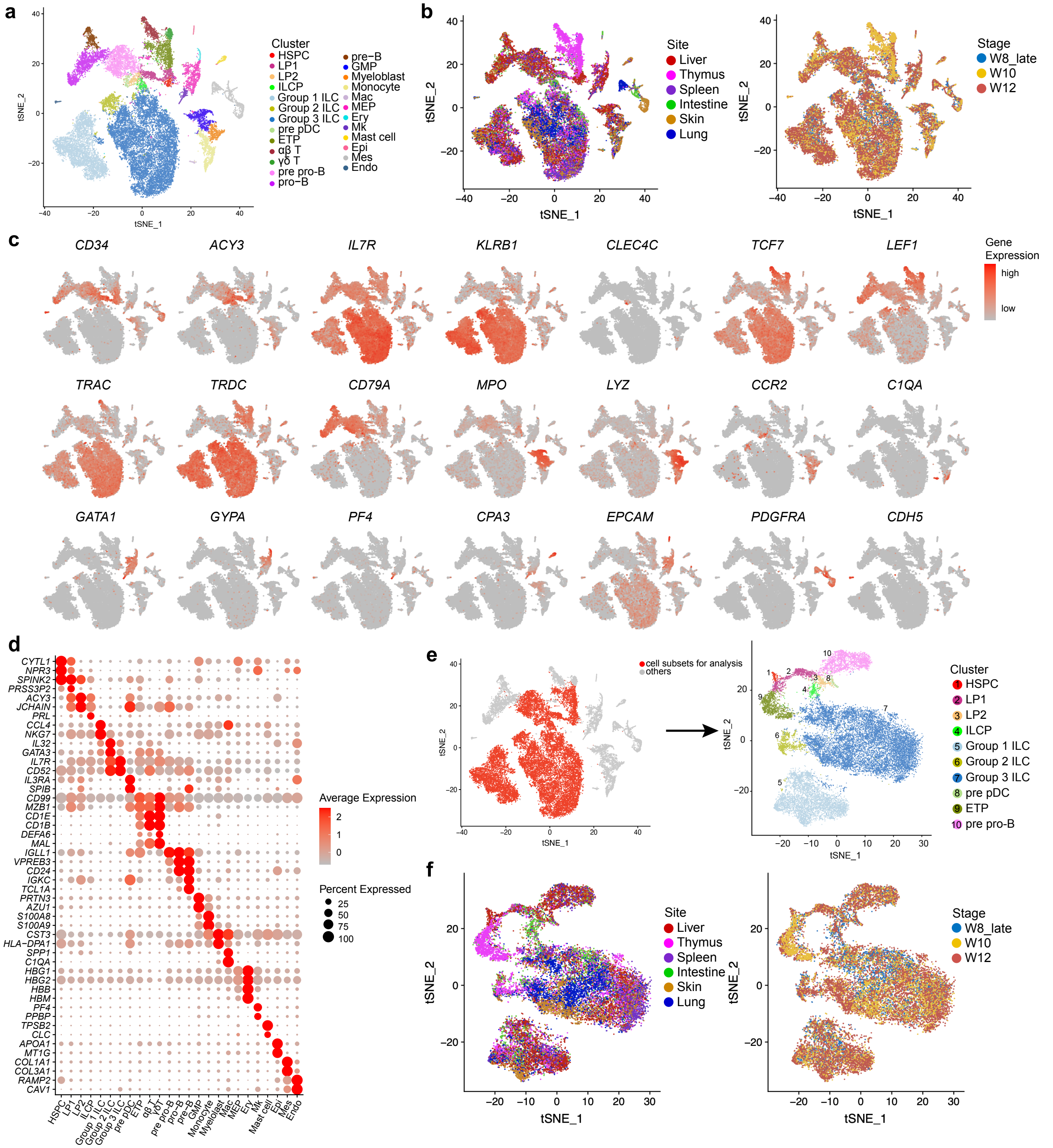
a
Cluster
HSPC
LP1
LP2
ILCP
Group 1 ILC
Group 2 ILC
Group 3 ILC
pre pDC
ETP
αβ T
γδ T
pre pro-B
pro−B
pre−B
GMP
Myeloblast
Monocyte
Mac
MEP
Ery
Mk
Mast cell
Epi
Mes
Endo
b
Site
Liver
Thymus
Spleen
Intestine
Skin
Lung
Stage
W8_late
W10
W12
c
CD34
ACY3
IL7R
KLRB1
CLEC4C
TCF7
LEF1
Gene Expression
high
low
TRAC
TRDC
CD79A
MPO
LYZ
CCR2
C1QA
GATA1
GYPA
PF4
CPA3
EPCAM
PDGFRA
CDH5
d
Average Expression
Percent Expressed
e
cell subsets for analysis
others
Cluster
HSPC
LP1
LP2
ILCP
Group 1 ILC
Group 2 ILC
Group 3 ILC
pre pDC
ETP
pre pro-B
f
Site
Liver
Thymus
Spleen
Intestine
Skin
Lung
Stage
W8_late
W10
W12
tSNE_1
tSNE_2

Supplementary Figure 5 (related to Figure 2) Overview of all cell clusters identified and lymphoid-related cell clusters were used for further analysis

(a) tSNE visualization of 25 cell clusters of combined data containing cells from human fetal hematopoietic (liver), lymphoid (thymus; spleen) and non-lymphoid (intestine; skin; lung) tissues of 6 samples colored by cell identity. **(b)** Site (left panel) and stage (right panel) information of cells are indicated by colors. **(c)** Expression of indicated featured genes projected onto tSNE. Colors indicate gene expression level. **(d)** Dot plots show the average expression level of top DEGs in each cluster. Colors represent the average expression and size encodes the proportion of gene-expressing cells. **(e)** 10 lymphoid-related cell clusters including HSPC, LP1, LP2, ILCP, Group 1 ILC, Group 2 ILC, Group 3 ILC, pre pDC, ETP, pre pro-B were used for further analysis. The highlighted color indicates lymphoid-related cell clusters used and the gray stands for others. **(f)** tSNE visualization of ten lymphoid-related cell clusters above, site (left panel) and stage (right panel) information indicated by colors.
