# Supplementary material for: Delineating spatiotemporal and hierarchical development of human fetal innate lymphoid cells
Source: Cell Res. 2021 Jul 8;31(10):1106–22. doi: 10.1038/s41422-021-00529-2 (PMC8486758; doi:10.1038/s41422-021-00529-2)
Supplement: Supplementary file 6 — Supplementary information, Fig. S6 [file 41422_2021_529_MOESM6_ESM.pdf]

Figure S6

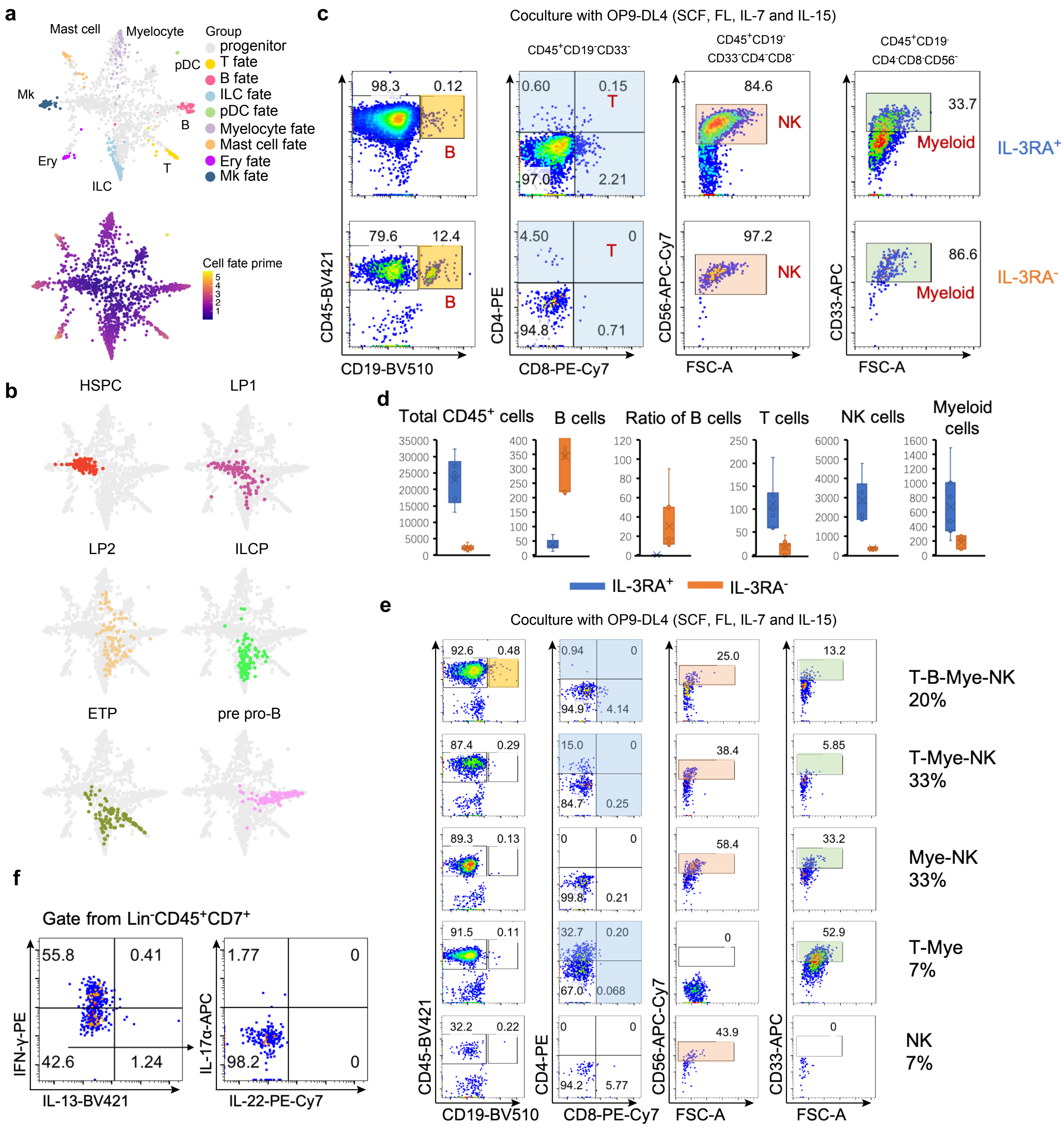

a
Mast cell
Myelocyte
pDC
Mk
B
Ery
T
ILC
Group
progenitor
T fate
B fate
ILC fate
pDC fate
Myelocyte fate
Mast cell fate
Ery fate
Mk fate
Cell fate prime
5
4
3
2
1
b
HSPC
LP1
LP2
ILCP
ETP
pre pro-B
c
Coculture with OP9-DL4 (SCF, FL, IL-7 and IL-15)
CD45+CD19-CD33-
CD45+CD19- CD33-CD4-CD8-
CD45+CD19- CD4-CD8-CD56-
98.3
0.12
B
0.60
0.15
T
97.0
2.21
84.6
NK
33.7
Myeloid
IL-3RA+
79.6
12.4
B
4.50
0
T
94.8
0.71
97.2
NK
86.6
Myeloid
IL-3RA-
CD45-BV421
CD19-BV510
CD4-PE
CD8-PE-Cy7
CD56-APC-Cy7
FSC-A
CD33-APC
FSC-A
d
Total CD45+ cells
B cells
Ratio of B cells
T cells
NK cells
Myeloid cells
IL-3RA+
IL-3RA-
e
Coculture with OP9-DL4 (SCF, FL, IL-7 and IL-15)
92.6
0.48
0.94
0
94.9
4.14
25.0
13.2
T-B-Mye-NK
20%
87.4
0.29
15.0
0
84.7
0.25
38.4
5.85
T-Mye-NK
33%
89.3
0.13
0
0
99.8
0.21
58.4
33.2
Mye-NK
33%
91.5
0.11
32.7
0.20
67.0
0.068
0
52.9
T-Mye
7%
32.2
0.22
0
0
94.2
5.77
43.9
0
NK
7%
CD45-BV421
CD19-BV510
CD4-PE
CD8-PE-Cy7
CD56-APC-Cy7
FSC-A
CD33-APC
FSC-A
f
Gate from Lin-CD45+CD7+
55.8
0.41
42.6
1.24
1.77
0
98.2
0
IFN-γ-PE
IL-13-BV421
IL-17α-APC
IL-22-PE-Cy7

Supplementary Figure 6 (related to Figure 3) In vitro bulk and clonal assay of IL-3RA$^+$ and IL-3RA$^-$ lymphoid progenitors

(**a**) Fully annotated STEMNET map visualizes the probabilities of progenitors to develop toward mature lineages including T, B, ILC, pDC (GEO: GSE143002), myelocyte, mast cell, erythrocyte and megakaryocyte. Colors indicate the mature cell types (upper panel) and the possibility of progenitor cells developing into the individual lineage (below panel). (**b**) Visualization of the probabilities of progenitor clusters in Fig. S6a to develop toward specific lineages. (**c)** IL-3RA$^+$ and IL-3RA$^-$ Lin-CD45$^+$CD34$^+$CD127$^+$ lymphoid progenitors (150 cells for each subset) were sorted into 24-well plate pre-seeded with OP9-DL4 and cocultured with SCF, FL, IL-7 and IL-15. The IL-3RA$^+$ lymphoid progenitors generate more CD45$^+$ hematopoietic cells including more T cells, NK and myeloid cells (upper panel) while IL-3RA$^-$ lymphoid progenitors show more B (below panel) lineage potential suggested by coculture experiments. T, B, NK and myeloid cells were identified as positive for its respective lineage markers and negative for other lineage markers. **(d)** Numbers of total CD45$^+$ cells, B cells, the percentage of B cells in total CD45$^+$ cells, T cells, NK cells as well as myeloid cells after co-cultured for 12 days on OP9-DL4 are shown in the bar graph. **(e,f)** Representative FACS

analysis of single cultured IL-3RA$^+$ Lin$^-$CD45$^+$CD34$^+$CD127$^+$ lymphoid progenitors on OP9-DL4 with SCF, FL, IL-7 and IL-15 **(e)** and under ILC-induction condition (IL-2, IL-7, IL-1β, IL-23, IL-25 and IL-33) **(f)** for 14 days.
