# Supplementary material for: Delineating spatiotemporal and hierarchical development of human fetal innate lymphoid cells
Source: Cell Res. 2021 Jul 8;31(10):1106–22. doi: 10.1038/s41422-021-00529-2 (PMC8486758; doi:10.1038/s41422-021-00529-2)
Supplement: Supplementary file 7 — Supplementary information, Fig. S7 [file 41422_2021_529_MOESM7_ESM.pdf]

Figure S7

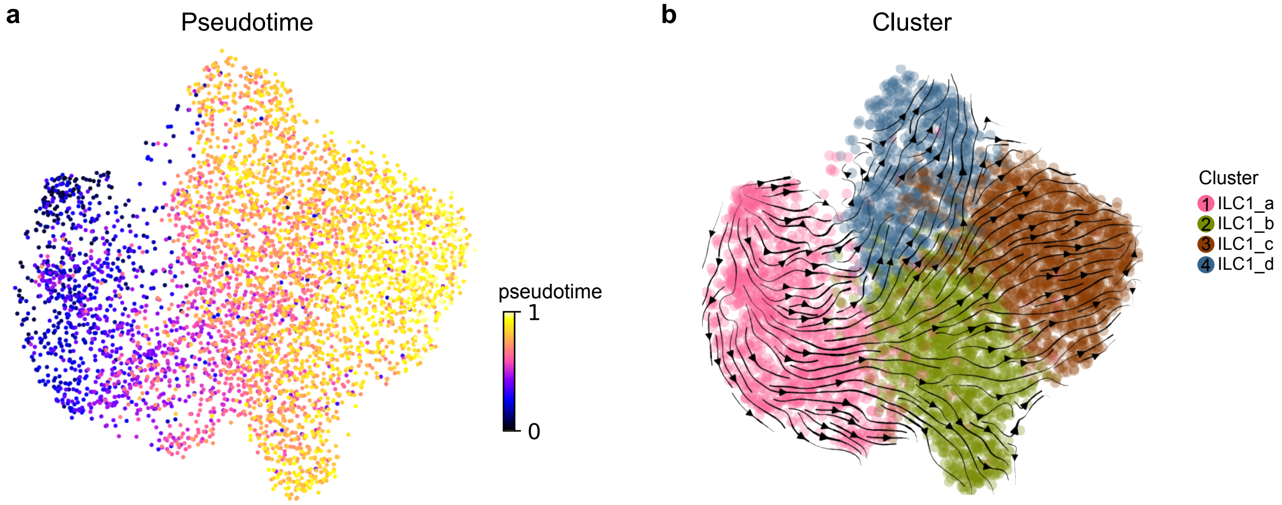
a
Pseudotime
pseudotime
1
0
b
Cluster
Cluster
1 ILC1_a
2 ILC1_b
3 ILC1_c
4 ILC1_d

Supplementary Figure 7 (related to Figure 4) Pseudotime analysis of ILC1 sub-clusters

pseudotemporal ordering (**a**) and directed transition probabilities (**b**) were evaluated by Cellrank based on KNN graph and CytoTRACE score derived from gene expression data and projected onto umap embedding.
