# Supplementary material for: Delineating spatiotemporal and hierarchical development of human fetal innate lymphoid cells
Source: Cell Res. 2021 Jul 8;31(10):1106–22. doi: 10.1038/s41422-021-00529-2 (PMC8486758; doi:10.1038/s41422-021-00529-2)
Supplement: Supplementary file 8 — Supplementary information, Fig. S8 [file 41422_2021_529_MOESM8_ESM.pdf]

Figure S8

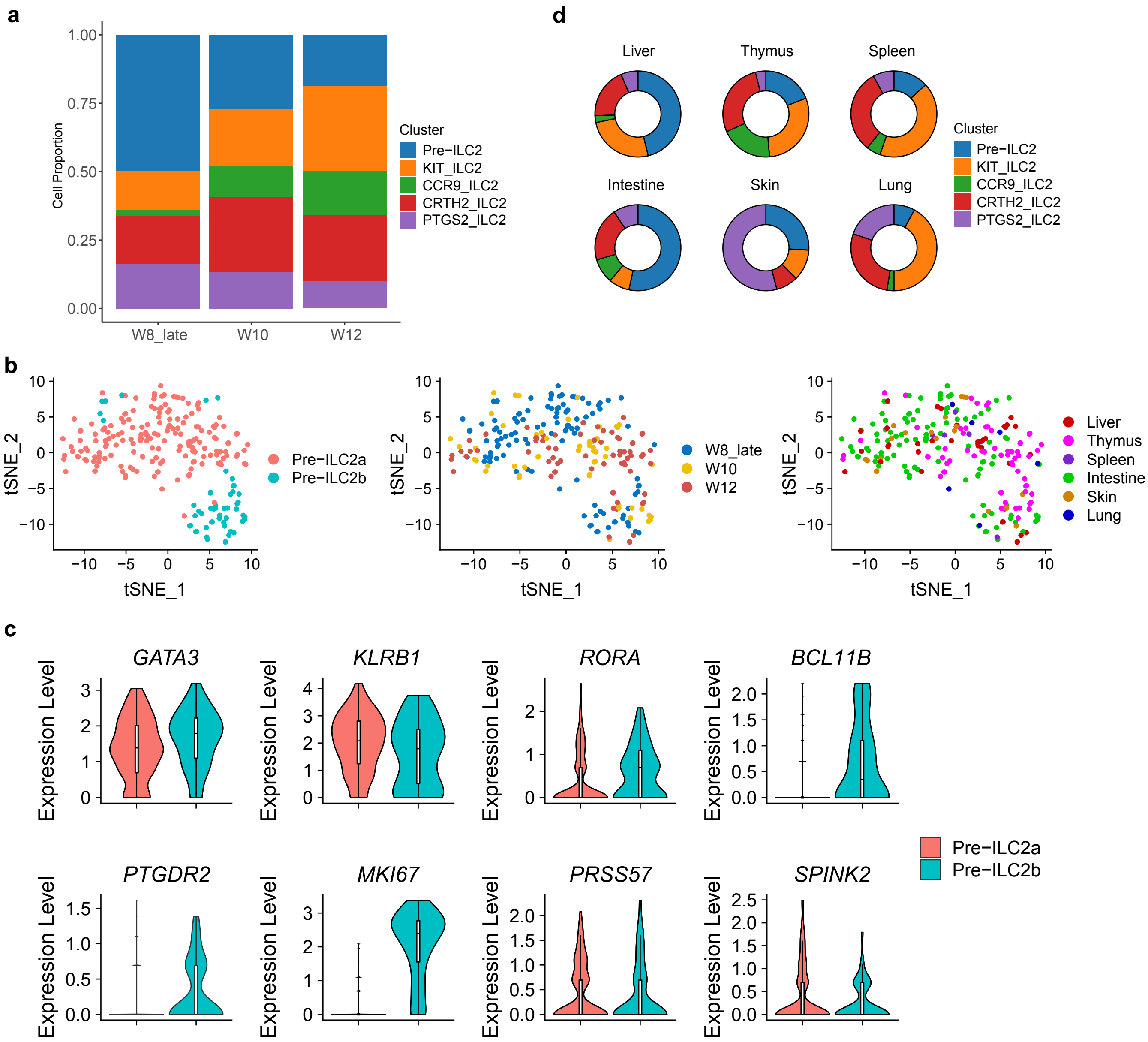

a
1.00
0.75
0.50
0.25
0.00
Cell Proportion
W8_late
W10
W12
Cluster
Pre−ILC2
KIT_ILC2
CCR9_ILC2
CRTH2_ILC2
PTGS2_ILC2
d
Liver
Thymus
Spleen
Intestine
Skin
Lung
Cluster
Pre−ILC2
KIT_ILC2
CCR9_ILC2
CRTH2_ILC2
PTGS2_ILC2
b
tSNE_2
tSNE_1
Pre−ILC2a
Pre−ILC2b
W8_late
W10
W12
Liver
Thymus
Spleen
Intestine
Skin
Lung
c
GATA3
KLRB1
RORA
BCL11B
PTGDR2
MKI67
PRSS57
SPINK2
Expression Level
Pre−ILC2a
Pre−ILC2b

Supplementary Figure 8 (related to Figure 5) Stage and tissue distribution of ILC2 sub-clusters

(a) Bar graphs show proportions of each ILC2 sub-cluster in different stages. Colors indicate different cell populations. **(b)** UMAP visualization of cells in Pre-ILC2 cluster are further separated into Pre-ILC2a and Pre-ILC2b subgroups by UMAP analysis (left panel). Stage (middle panel) and site (right panel) information of Pre-ILC2 subgroups is indicated by colors. (**c**) Violin plots show the expression level of indicated genes in Pre-ILC2a and Pre-ILC2b subgroups. Colors indicate subgroups. **(d)** Pie charts show the tissue distribution of each ILC2 sub-cluster. Colors indicate different cell populations.
