# Supplementary material for: Delineating spatiotemporal and hierarchical development of human fetal innate lymphoid cells
Source: Cell Res. 2021 Jul 8;31(10):1106–22. doi: 10.1038/s41422-021-00529-2 (PMC8486758; doi:10.1038/s41422-021-00529-2)
Supplement: Supplementary file 9 — Supplementary information, Fig. S9 [file 41422_2021_529_MOESM9_ESM.pdf]

Figure S9

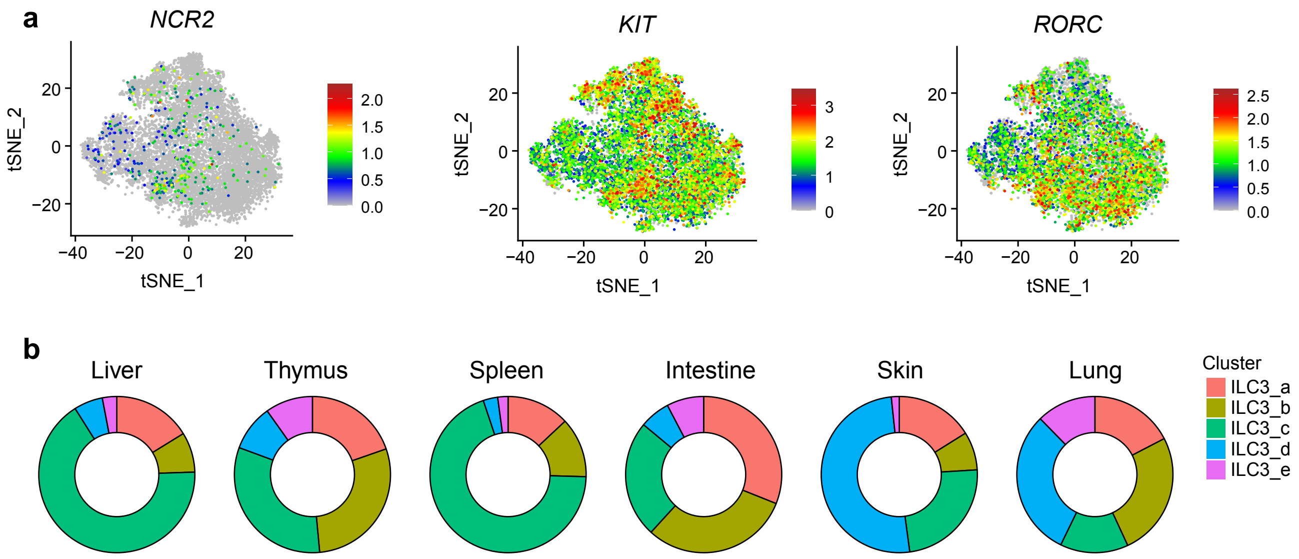
a
NCR2
KIT
RORC
tSNE_1
tSNE_2
b
Liver
Thymus
Spleen
Intestine
Skin
Lung
Cluster
ILC3_a
ILC3_b
ILC3_c
ILC3_d
ILC3_e

Supplementary Figure 9 (related to Figure 6) Tissue distribution of ILC3 sub-clusters

(a) Expression of indicated curated ILC3-related genes projected onto UMAP of ILC3. Colors indicate the expression levels. **(b)** Pie charts show tissue distribution of ILC3 sub-cluster. Colors indicate different cell populations.
