# Supplementary material for: Delineating spatiotemporal and hierarchical development of human fetal innate lymphoid cells
Source: Cell Res. 2021 Jul 8;31(10):1106–22. doi: 10.1038/s41422-021-00529-2 (PMC8486758; doi:10.1038/s41422-021-00529-2)
Supplement: Supplementary file 10 — Supplementary information, Fig. S10 [file 41422_2021_529_MOESM10_ESM.pdf]

Figure S10

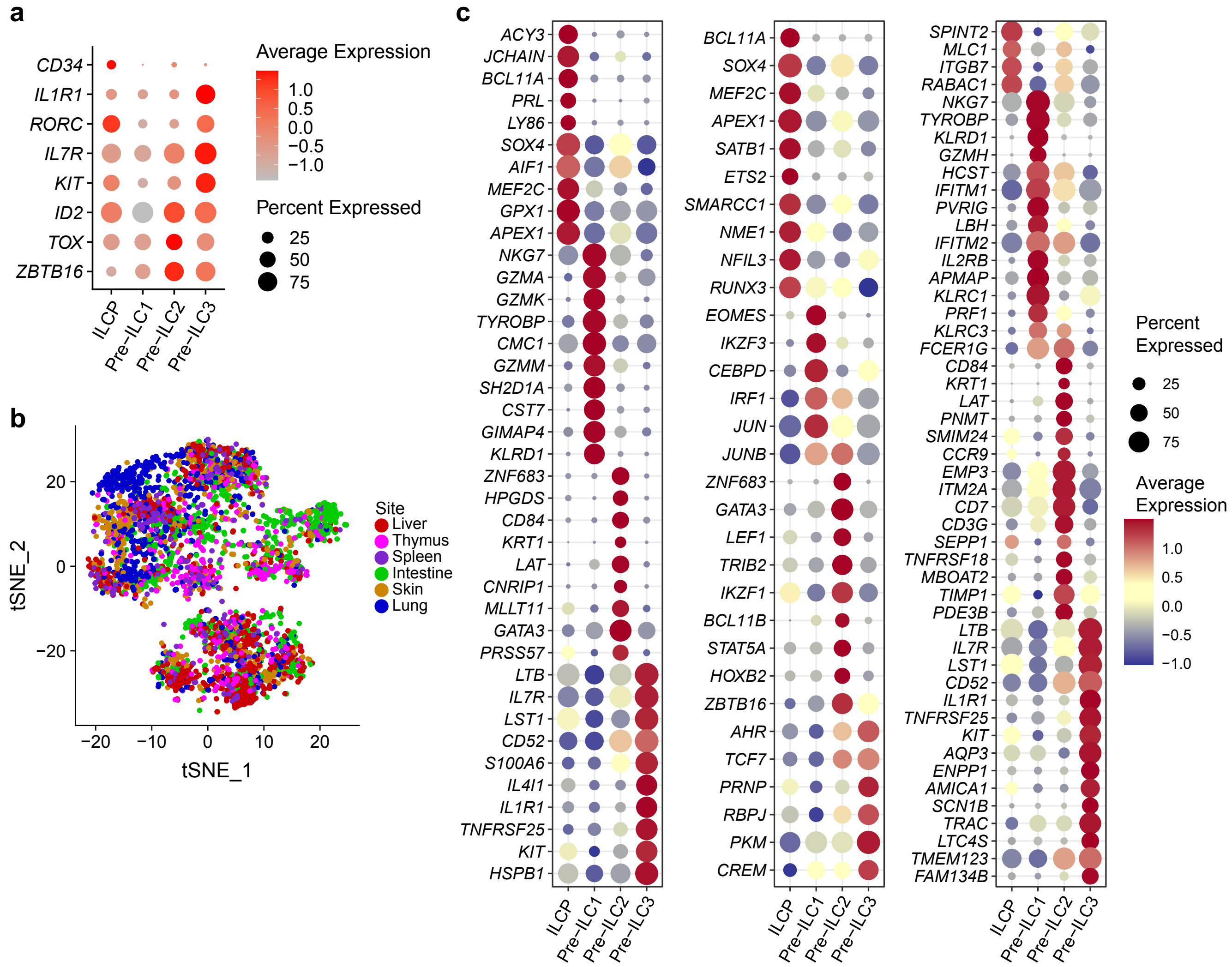

a
CD34
IL1R1
RORC
IL7R
KIT
ID2
TOX
ZBTB16
ILCP
Pre−ILC1
Pre−ILC2
Pre−ILC3
Average Expression
1.0
0.5
0.0
−0.5
−1.0
Percent Expressed
25
50
75
b
tSNE_2
tSNE_1
20
0
−20
−20
−10
0
10
20
Site
Liver
Thymus
Spleen
Intestine
Skin
Lung
c
ACY3
JCHAIN
BCL11A
PRL
LY86
SOX4
AIF1
MEF2C
GPX1
APEX1
NKG7
GZMA
GZMK
TYROBP
CMC1
GZMM
SH2D1A
CST7
GIMAP4
KLRD1
ZNF683
HPGDS
CD84
KRT1
LAT
CNRIP1
MLLT11
GATA3
PRSS57
LTB
IL7R
LST1
CD52
S100A6
IL4I1
IL1R1
TNFRSF25
KIT
HSPB1
BCL11A
SOX4
MEF2C
APEX1
SATB1
ETS2
SMARCC1
NME1
NFIL3
RUNX3
EOMES
IKZF3
CEBPD
IRF1
JUN
JUNB
ZNF683
GATA3
LEF1
TRIB2
IKZF1
BCL11B
STAT5A
HOXB2
ZBTB16
AHR
TCF7
PRNP
RBPJ
PKM
CREM
SPINT2
MLC1
ITGB7
RABAC1
NKG7
TYROBP
KLRD1
GZMH
HCST
IFITM1
PVRIG
LBH
IFITM2
IL2RB
APMAP
KLRC1
PRF1
KLRC3
FCER1G
CD84
KRT1
LAT
PNMT
SMIM24
CCR9
EMP3
ITM2A
CD7
CD3G
SEPP1
TNFRSF18
MBOAT2
TIMP1
PDE3B
LTB
IL7R
LST1
CD52
IL1R1
TNFRSF25
KIT
AQP3
ENPP1
AMICA1
SCN1B
TRAC
LTC4S
TMEM123
FAM134B
ILCP
Pre−ILC1
Pre−ILC2
Pre−ILC3
Percent Expressed
25
50
75
Average Expression
1.0
0.5
0.0
−0.5
−1.0

**Supplementary Figure 10 (related to figure 7) Differentially expressed genes in ILCP and three Pre-ILCs**

**(a)** Dot plots show the average expression level of reported feature genes in ILCP, Pre-ILC1, Pre-ILC2 and Pre-ILC3 clusters. Colors represent the average expression, and size encodes the proportion of gene-expressing cells. **(b)** Tissue distribution of UMAP of ILCPs and three Pre-ILC cells. Colors indicate different tissues. **(c)** Dot plots show the average expression level of top 10 DEGs (right panel), TF-encoding (middle panel) and surface marker encoding (right panel) genes in ILCP and 3 Pre-ILC clusters. Colors represent the average expression, and size encodes the proportion of gene-expressing cells.
